# Evaluation of the cytotoxicity of the Bithionol-paclitaxel combination in a panel of human ovarian cancer cell lines

**DOI:** 10.1371/journal.pone.0185111

**Published:** 2017-09-20

**Authors:** Vijayalakshmi N. Ayyagari, Paula L. Diaz-Sylvester, Tsung-han Jeff Hsieh, Laurent Brard

**Affiliations:** 1 Division of Gynecologic Oncology, Department of Obstetrics and Gynecology, Southern Illinois University School of Medicine, Springfield, IL, United States of America; 2 Center for Clinical Research, Southern Illinois University School of Medicine, Springfield, IL, United States of America; 3 Simmons Cancer Institute at SIU, Southern Illinois University School of Medicine, Springfield, IL, United States of America; University of South Alabama Mitchell Cancer Institute, UNITED STATES

## Abstract

Previously, Bithionol (BT) was shown to enhance the chemosensitivity of ovarian cancer cell lines to cisplatin treatment. In the present study, we focused on the anti-tumor potential of the BT-paclitaxel combination when added to a panel of ovarian cancer cell lines. This *in vitro* study aimed to 1) determine the optimum schedule for combination of BT and paclitaxel and 2) assess the nature and mechanism(s) underlying BT-paclitaxel interactions. The cytotoxic effects of both drugs either alone or in combination were assessed by presto-blue cell viability assay using six human ovarian cancer cell lines. Inhibitory concentrations to achieve 50% cell death (IC_50_) were determined for BT and paclitaxel in each cell line. Changes in levels of cleaved PARP, XIAP, bcl-2, bcl-xL, p21 and p27 were determined via immunoblot. Luminescent and colorimetric assays were used to determine caspases 3/7 and autotaxin (ATX) activity. Cellular reactive oxygen species (ROS) were measured by flow cytometry. Our results show that the efficacy of the BT-paclitaxel combination depends upon the concentrations and sequence of addition of paclitaxel and BT. Pretreatment with BT followed by paclitaxel resulted in antagonistic interactions whereas synergistic interactions were observed when both drugs were added simultaneously or when cells were pretreated with paclitaxel followed by BT. Synergistic interactions between BT and paclitaxel were attributed to increased ROS generation and enhanced apoptosis. Decreased expression of pro-survival factors (XIAP, bcl-2, bcl-xL) and increased expression of pro-apoptotic factors (caspases 3/7, PARP cleavage) was observed. Additionally, increased expression of key cell cycle regulators p21 and p27 was observed. These results show that BT and paclitaxel interacted synergistically at most drug ratios which, however, was highly dependent on the sequence of the addition of drugs. Our results suggest that BT-paclitaxel combination therapy may be effective in sensitizing ovarian cancer cells to paclitaxel treatment, thus mitigating some of the toxic effects associated with high doses of paclitaxel.

## Introduction

It is reported that less than 33% of ovarian cancer patients respond to current second or third-line chemotherapy due to development of drug resistance [1–4]. This necessitates new or alternate options for second-line therapy to overcome drug resistance and to enhance the efficacy of drugs for use in patients with drug-resistant ovarian cancer. Paclitaxel (also known as Taxol) is an effective chemotherapeutic agent against drug-resistant breast and ovarian cancers [5]. It has shown promising clinical efficacy against such cancers in both first and second line treatment regimens. Paclitaxel is an effective mitotic inhibitor which plays an important role in the formation and stabilization of microtubules resulting in cell cycle block at the metaphase to anaphase transition, thus inducing cytotoxicity [6, 7]. Although most patients respond initially to paclitaxel treatment, the subsequent therapeutic failure of this drug was attributed to the development of drug resistance [8, 9]. Dose induced toxicity and side effects are the other common problems associated with paclitaxel treatment [10, 11]. Similarly, the tolerability of *combination* treatment with paclitaxel and cisplatin is also limited due to the development of neuropathies and neurotoxicity [10, 12]. These disadvantages make a strong case for the use of these drugs only at low concentrations, thus necessitating the need for combination with other drugs or chemo-sensitizers/drug-resistance modulators in order to enhance efficacy, overcome drug resistance and/or mitigate/eliminate toxic side effects. It is essential to explore other drugs which can target alternative/similar pathways to paclitaxel or cisplatin without toxic effects, thus providing alternate therapeutic options for ovarian cancer patients. Reports of compounds used in combination with cisplatin or paclitaxel have been published, however, none of these combinations were sufficiently effective for use in the clinic [13, 14]. In this study, we investigated the novel approach of combining paclitaxel with Bithionol [2, 2’-Sulfanediylbis (4, 6-dichlorophenol)] (BT). BT, is an anti-parasitic drug approved by the Food and Drug Administration for human use as a second-line oral medication for the treatment of helminthic infections [15]. Previously, we showed the anti-tumor potential of BT in an *in vitro* study using a panel of ovarian cancer cell lines with varying cisplatin sensitivities [16]. BT concentrations for half maximal inhibition (IC_50_) values were well below the clinically tolerable doses. We also showed that BT enhanced the sensitivity of cisplatin resistant cell lines to cisplatin by enhancing ROS generation and by altering the expression of key apoptotic regulators [16]. In a separate study, BT was also shown to induce apoptosis *in vivo* without toxicity at any of the doses tested [17]. Additionally, our recent *in vitro* study showed that when added in combination with cisplatin, BT can be either synergistic or antagonistic depending on the concentration ratio, implying the need to administer these drugs at optimal doses [18]. To further explore the anti-tumor potential of BT, it is critical to understand the nature of the interaction(s) between BT and paclitaxel. In this study, we assessed the antitumor efficacy of BT in combination with paclitaxel using a panel of ovarian cancer cell lines with varying cisplatin sensitivities. The major objectives of the present study were to determine optimal combination of BT and paclitaxel to achieve maximum cytotoxic activity and to understand the mechanism(s) of action of the BT-paclitaxel combination. BT and paclitaxel combinations were investigated systematically for drug-ratio dependent interactions *in vitro*. Using an approach similar to our previous study [18], the nature of the interactions between BT and paclitaxel were evaluated using three different methodologies: (1) pretreatment with BT for 24 hours followed by paclitaxel addition (sequential addition in non-constant ratio), (2) pretreatment with paclitaxel for 24 hours followed by BT addition (sequential addition in a non-constant ratio), and (3) simultaneous addition of both drugs in a non-constant ratio. The combination index was used to evaluate if BT-paclitaxel interactions were antagonistic, synergistic or additive. ROS generation, autotaxin inhibition, induction of apoptosis and expression of key apoptotic and cell cycle modulators were evaluated to elucidate BT-paclitaxel combination mechanism(s) of action.

## Materials and methods

### Cell lines and chemicals

Human ovarian carcinoma cell lines, OVACAR-3, SKOV-3 were provided by Dr. McAsey (SIU School of Medicine, Springfield, IL). Isogenic ovarian cancer cell lines pairs, e.g., A2780 /A2780-CDDP and IGROV-1/, IGROV-1CDDP were received as a generous gift from Dr. Brodsky (Brown University, Providence, RI). The parental cell lines were purchased from Sigma and made resistant *in vitro* by continuous stepwise exposure to cisplatin to produce the corresponding cisplatin-resistant cell lines. All cell lines were maintained in DMEM media (Sigma) as described previously [16]. The cisplatin-resistant variants cells were treated with 3 μM cisplatin every third passage to maintain cisplatin resistance.

Bithionol (2, 2’-Sulfanediylbis (4, 6-dichlorophenol)), BT, and paclitaxel were purchased from Sigma (St Louis, MO). All primary antibodies were purchased from Cell Signaling Technologies, (Danvers, MA). PrestoBlue™ Cell Viability Reagent and ROS Dye– 5, 6-carboxy-2′, 7′-dichlorodihydrofluorescein diacetate (carboxy-H_2_DCFDA or, C400) were purchased from Invitrogen (Carlsbad, CA).

### Cell viability assay

Cell viability after drug(s) treatment for 48 hours was determined by Presto Blue cell viability reagent (Invitrogen) as descried previously [16]. BT was tested at concentrations ranging from 3.56 μM to 100 μM and paclitaxel at concentrations between 0.98 nM and 31.5 nM. DMSO concentration was corrected to 1% in all wells. Vehicle-treated control cells (media with 1% DMSO) were considered as 100% viable against which treated cells were compared to. Data were expressed as mean ± SD of triplicate experiments.

In order to determine role of ROS in BT-paclitaxel induced cytotoxicity, we performed cell viability assays in the presence of the antioxidant ascorbic acid (AA). Cells were pretreated with 1 mM AA for 2 hours before prior to the addition of drugs and further incubated for 48 hours with the tested drugs and 1 mM AA. Restoration of cell viability was assessed by comparing cell viability in presence or absence of AA [16]. For all studies, triplicate wells were set up for each treatment condition. Data were expressed as mean ± SD of triplicate experiments.

### Dose response curves for IC_50_ determination

Dose response curves were generated for paclitaxel and BT to determine the inhibitory concentration to achieve 50% cell death (IC_50_ values) for each human ovarian cancer cell lines (OVCAR-3, SKOV-3, A2780, A2780-CDDP, IGROV-1 & IGROV-1-CDDP), as described previously [16]. Briefly, cells were treated with different concentrations of BT and paclitaxel for 48 hours at concentrations ranging from 0.178 μM to 400 μM for BT and 0.98 nM to 100 nM for paclitaxel. BT stock (20 mM) and paclitaxel stock (10 mM) were prepared in DMSO. For both drugs, all the working dilutions were prepared in DMEM media. All experiments were done in triplicate. Dose response curves to calculate IC_50_ values were plotted using Graph Pad Prism Software.

### Drug combination studies

To assess combination effects of BT with paclitaxel, these drugs were combined in non-constant ratios in which BT and paclitaxel were prepared at a series of concentrations that span the dose-response curves for both drugs. Each concentration of BT was mixed with each concentration of paclitaxel, thereby producing a matrix of multiple stock admixtures, containing both drugs together in solution at a variety of concentrations and ratios. The dose ranges selected for combination studies were 1.56 μM to 200 μM for BT and 0.98 nM to 31.25 nM for paclitaxel.

The nature of the interaction between BT and paclitaxel was assessed using three different scheduling approaches: (1) simultaneous treatment with both drugs, where cells were treated with both BT and paclitaxel simultaneously combined in a non-constant ratio, for 48 hours; (2) pretreatment with paclitaxel followed by addition of BT in a non-constant ratio, where cells were treated with different concentrations of paclitaxel for 24 hours after which paclitaxel was removed and BT was added for another 24 hours and (3) pretreatment with BT followed by addition of paclitaxel in a non-constant ratio, where cells were treated with different concentrations of BT for 24 hours after which BT was removed and paclitaxel was added for another 24 hours.

The cytotoxic potential of BT-paclitaxel combination over a range of concentrations was compared to that obtained for the individual drugs, and a measure of the synergy between the two drugs, referred to as the combination index (CI), was calculated using a median-effect mathematical algorithm according to Chou’s methods [19]. CalcuSyn (BioSoft) was used to calculate CI values for drug combinations. A drug combination is synergistic if its CI value is significantly below 1; the combination is additive where the CI is between 0.9 and 1.0; and the combination is antagonistic as indicated by CI values above 1.0. Triplicate wells were set up for each treatment condition. All the experiments were performed in triplicates and data represents mean ± SD.

### Caspase 3/7 assay

Caspase 3/7 activity was measured using Caspase-Glo 3/7 assay kit from Promega, as described previously [16]. Briefly, 10^4^ cells were treated with BT and paclitaxel either alone or in combination. Cells were treated with individual drugs at concentrations at which 60–80% cells are still viable. Following treatment for 48 hours, Caspase-Glo 3/7 reagent was added and incubated for 30 min. at room temperature. The luminescence intensity was measured using a luminometer (luminoskan, Thermo Scientifics). Cells treated with vehicle (1% DMSO media) were considered as control against which treated cells were compared. Triplicate wells were set up for each treatment condition. Data were expressed as mean ± SD of triplicate experiments.

### Apoptosis detection via Hoechst staining

To detect nuclear condensation indicative of apoptosis, NucBlue Live Cell Stain (Hoechst 33342) was used (Invitrogen, Carlsbad, CA). Hoechst staining was performed as described previously [16, 20]. For A2780 and A2780-CDDP, cells were treated with 12.5 μM BT and 4 nM paclitaxel either alone or in combination for 48 hours. Similarly, IGROV-1 and IGROV-1-CDDP cells were treated with 50 μM BT or 4 nM paclitaxel for 48 hours either alone or in combination. Following treatment, cells were washed stained with Hoechst stain (2 drops/mL of media) for 15 min. at 25°C and observed under a fluorescent microscope. Representative images were taken with an inverted microscope (Olympus H4-100, CCD camera) and 20× objective. Duplicate wells were set up for each treatment condition. Each experiment was repeated three times. After morphological assessment by nuclear staining, the extent of apoptosis was quantified using the TUNEL assay.

### TUNEL assay

DNA fragmentation was detected using the TiterTACS® 2 TdT i*n Situ* Colorimetric Apoptosis Detection Kit (Trevigen, Gaithersburg, MD) following the manufacturer’s instructions. For A2780 and A2780-CDDP, cells were treated with 12.5 μM BT and 4 nM paclitaxel either alone or in combination for 48 hours. Similarly, IGROV-1 and IGROV-1-CDDP cells were treated with 50 μM BT or 4 nM paclitaxel for 48 hours, either alone or in combination. Following treatment for 48 hours, cells were washed and fixed followed by addition of labeled nucleotides and subsequent detection with HRP–HRP substrate (TACS-Sapphire) system. The absorbance was measured at 450 nm using a microplate reader, Multiskan (Thermo Scientifics). Triplicate wells were set up for each treatment condition. Data were expressed as mean ± SD of triplicate experiments.

### Estimation of ROS production

Hydrogen peroxide, hydroxyl radicals and peroxy radicals were detected via carboxy-H2DCFDA which is a chemically reduced, acetylated form of fluorescein. The assay was carried out as per the manufacturer’s instructions (Invitrogen, Carlsbad, CA). Briefly, cells (1×10^6^) were treated with BT and paclitaxel either alone or in combination. Cells were treated with individual drugs at concentrations at which 60–80% cells are viable. After treatment for 48 hours, the cells were incubated with 5 μM carboxy-H_2_DCFDA for 30 min at 37°C and fluorescence measured (490 nm excitation/520 nm emissions) using a florescence plate reader. Triplicate wells were set up for each treatment condition. Data were expressed as mean ± SD of triplicate experiments.

### Western blot analysis

Western blotting was carried out to analyze expression of key modulators of apoptosis, i.e. cleaved PARP, XIAP, bcl-2 and bcl-xL. Key cell cycle regulators such as p21 and p27 were also assessed by western blotting. Cell seeding, cell lysis and western botting were performed as described previously [16]. Briefly, cells were treated with BT and paclitaxel either alone or in combination. Cells were treated with individual drugs at concentrations at which 60–80% cells are viable. Following treatment for 48 hours, cells were harvested and lysed in cell extraction buffer (Invitrogen, Carlsbad, CA) containing 10 mM Tris, pH 7.4, 100 mM NaCl, 1 mM EDTA, 1 mM EGTA, 1 mM NaF, 20 mM Na_4_P_2_O_7_, 2 mM Na_3_VO_4_, 1% Triton X-100, 10% glycerol, 0.1% SDS, 0.5% deoxycholate protease inhibitor cocktail and PMSF. Cell lysates were subjected to western blotting. Following overnight incubation with primary antibodies at 4°C and subsequent incubation with appropriate secondary antibodies (Licor), the proteins on the blots were detected using a Licor image analyzer system. Each experiment was repeated three times.

### Autotaxin (ATX) assay

The phosphodiesterase activity of ATX was measured as described previously [16]. Briefly, cells were treated with individual drugs at concentrations at which 60–80% cells are viable. Following treatment for 48 hours, cell-free supernatants were collected. The concentration of ATX was normalized with respect to the cell mass of samples in each well. To estimate ATX, 100 µL cell-free culture media were incubated with 100 μL substrate containing *p*-nitrophenylphosphonate (pNppp) at a final concentration of 5 mM prepared in 50 mM Tris-HCl buffer, pH 9.0. After 30 min. of incubation at 37°C, the reaction was stopped by the addition of 100 μL of 0.1 M NaOH solution. The reaction product was measured by reading the absorbance at 410 nm. ATX inhibition of treated cells was calculated as the percentage of ATX activity in comparison with untreated cells. Triplicate wells were set up for each treatment condition. Data were expressed as mean ± SD of triplicate experiments.

### Statistical analysis

Comparisons between paclitaxel treated and BT/paclitaxel combination treated groups were performed by Student’s t-test. The significance level was set at p < 0.05.

## Results

### Dose response curves for IC50 determination

BT and paclitaxel induced cell death in a time and dose dependent manner when added alone for 48 hours. The IC_50_ values ranged from 25 ± 2 to 94 ± 16 μM for BT (data attached as supporting information: S1 Table & S1 Fig) and 4 ± 1 to 13.2 ± 1 nM for paclitaxel.

#### BT-paclitaxel combination cytotoxicity studies

**Fig 1:**
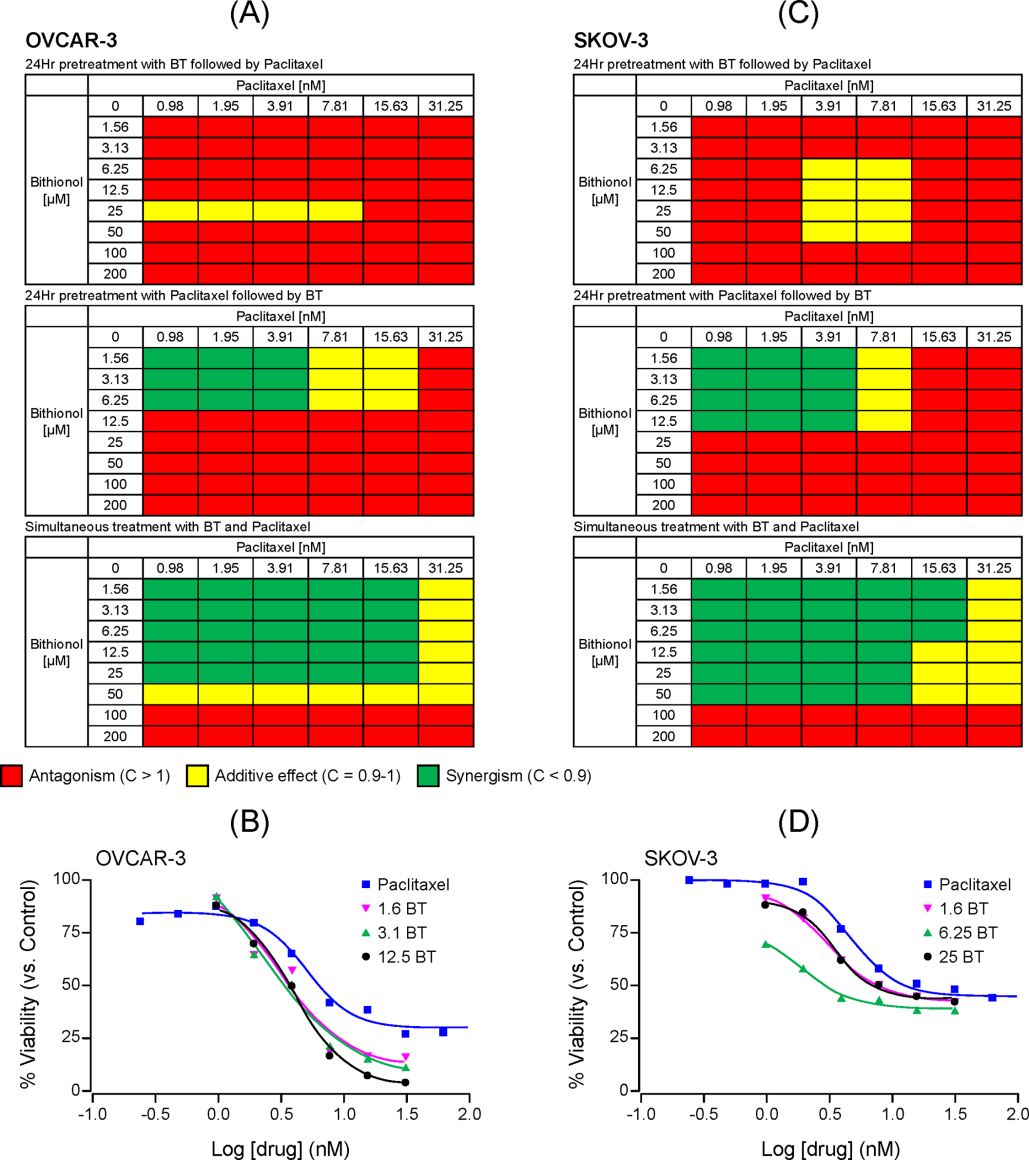
Cytotoxic potential of BT-paclitaxel combination on the cancer cell lines OVCAR-3 and SKOV-3. Combination index (CI) values were calculated based on the cytotoxicity data and presented as heat maps where a drug combination is synergistic (green color) if CI < 0.9; additive (yellow color) if CI is between 0.9 and 1.0; and antagonistic (red color) if CI > 1.0. Combination index values for OVCAR-3 and SKOV-3 are shown in **(A)** and **(C),** respectively. Dose response curves for OVCAR-3 **(B)** and SKOV-3 **(D)** when treated with paclitaxel alone or BT-paclitaxel combination (simultaneous addition). All experiments were performed in triplicate.

**Fig 2:**
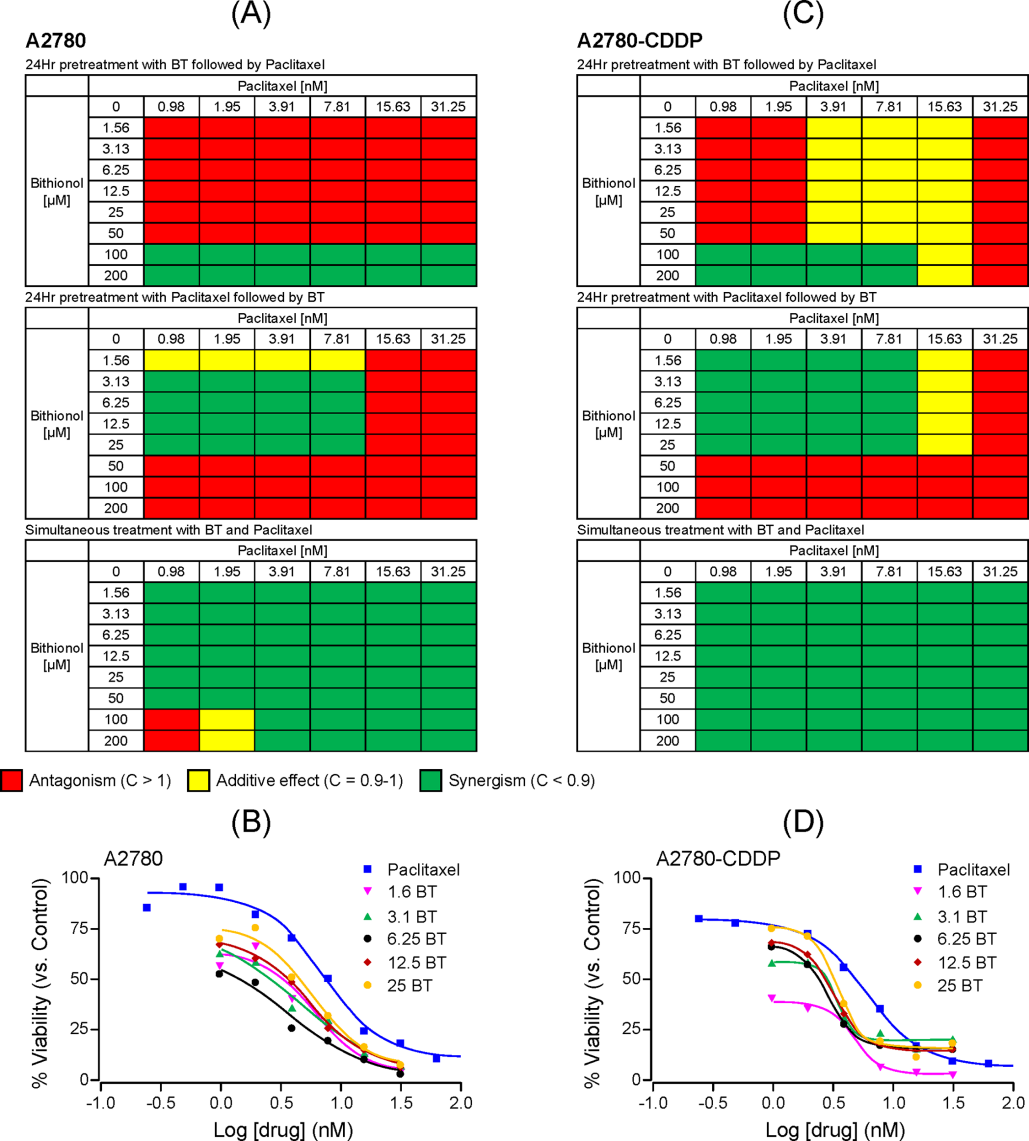
Cytotoxic potential of BT-paclitaxel combination on the isogenic pair of ovarian cancer cell lines A2780 (cisplatin-sensitive) and A2780-CDDP (cisplatin-resistant). Combination index (CI) values were calculated based on the percentage viability of cells treated with combinations of BT and paclitaxel. The nature of BT-paclitaxel interactions is represented in heat maps where synergism is shown in green (if CI < 0.9); additive effects are shown in yellow color (if CI is between 0.9 and 1.0); and antagonism is shown in red (if CI > 1.0). Combination index values for A2780 and A2780-CDDP are shown in **(A)** and **(C),** respectively. Dose response curves for A2780 **(B)** and A2780-CDDP **(D),** respectively when treated with paclitaxel alone or BT-paclitaxel combination (simultaneous addition). All experiments were performed in triplicate.

**Fig 3:**
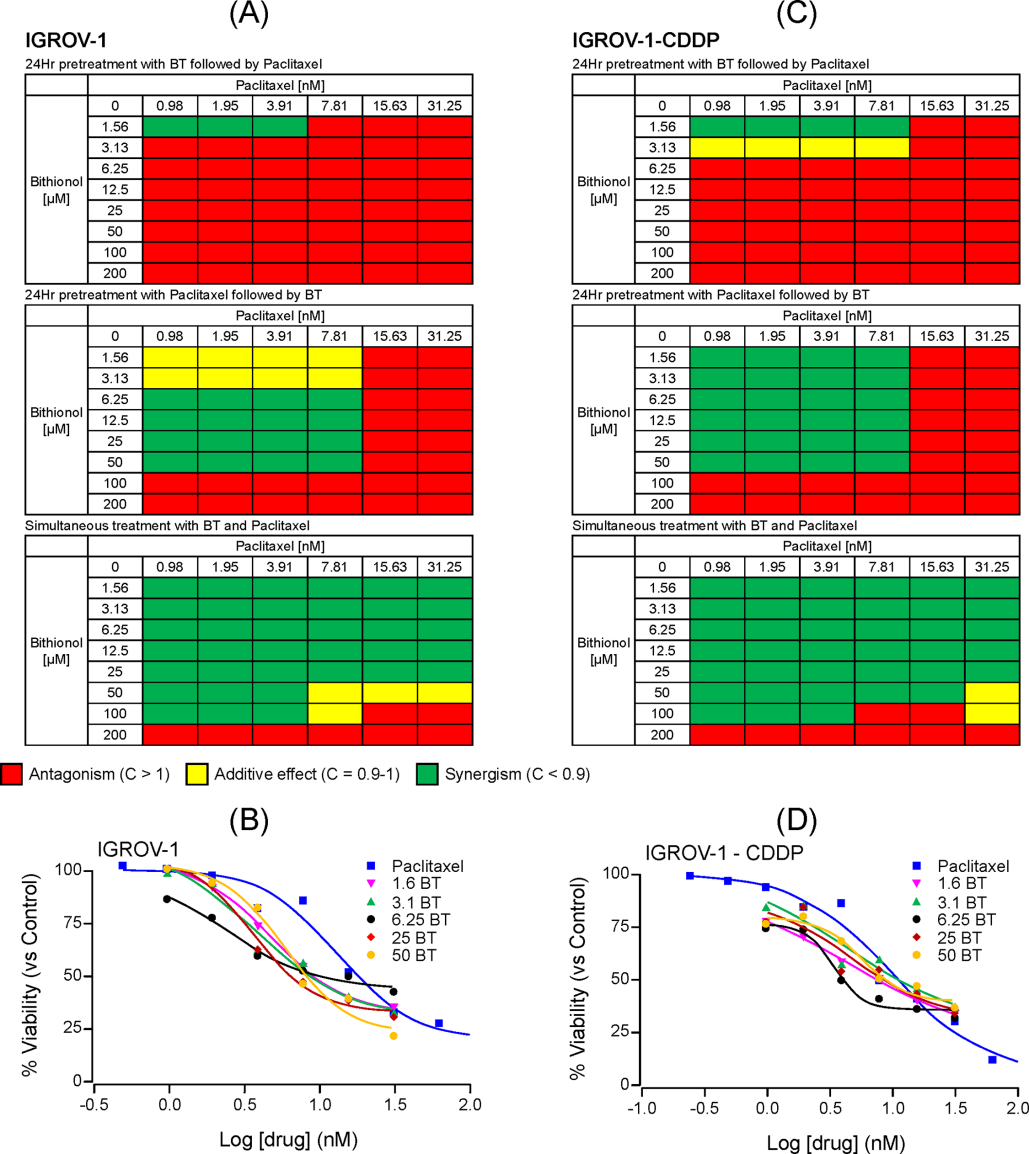
Cytotoxic potential of BT-paclitaxel combination on the isogenic pair of ovarian cancer cell lines IGROV-1 (cisplatin-sensitive) and IGROV-1-CDDP (cisplatin-resistant). *C*ombination index (CI) values were calculated and represented as heat maps where a drug combination is synergistic (green color) if CI < 0.9; additive (yellow color) if CI is between 0.9 and 1.0; and antagonistic (red color) if CI > 1.0. **(A)** and **(C)** show CI values for IGROV-1 and IGROV-1-CDDP, respectively. Dose response curves for IGROV-1 **(B)** and IGROV-1-CDDP **(D)** when treated with paclitaxel alone or BT/paclitaxel combination (simultaneous addition). All experiments were performed in triplicate.

The cytotoxicity of BT-paclitaxel combination was compared to that of each agent alone to evaluate for potential changes in IC_50_ value. The combination index (CI) results are represented in a heat map where synergism (CI < 1) is shown in green, additive effect (CI = 1) is shown in yellow and antagonism (CI > 1) is shown in red (Figs 1–3). BT-paclitaxel combination-induced cytotoxicity profiles on individual ovarian cancer cell lines are described below:

#### OVCAR-3

We observed that BT was antagonistic to paclitaxel when cells were pretreated with BT followed by paclitaxel. However, pretreatment with paclitaxel followed by BT resulted in synergism which was dependent on the concentrations of both drugs. Synergism was observed only at lower concentrations of BT and paclitaxel. Interestingly, simultaneous addition of BT with paclitaxel was found to be synergistic, especially at lower BT concentrations (1.56–25 μM) (Fig 1A). The IC_50_ value for BT was 57 ± 8 μM whereas the IC_50_ value for paclitaxel alone was 5.05 ± 1nM which decreased to 3.1 ± 0.6, 2.2 ± 0.5 and 3.8 ± 0.99 nM upon combination with BT at 1.56, 3.25 and 12.5 μM concentrations, respectively (Fig 1B). Higher concentration of BT antagonized the cytotoxic effects of paclitaxel regardless of the sequence of addition of drugs. In summary, BT and paclitaxel display synergy in OVCAR-3 cells that is dependent on drug concentrations and the sequence of addition.

#### SKOV-3

Similar to OVCAR-3, when cells were pretreated with BT followed by paclitaxel, the drugs were antagonistic at most concentrations of BT and paclitaxel. Interestingly, an additive effect was observed at specific concentrations of paclitaxel (3.91–7.81nM) and BT (6.25–50 μM). Pretreatment with paclitaxel followed by BT resulted in synergism only at lower concentrations of BT and paclitaxel. In contrast, when added simultaneously, BT was synergistic to paclitaxel except for the highest concentrations of BT and paclitaxel (Fig 1C). The IC_50_ value for BT was 58 ± 3 μM whereas the IC_50_ value for paclitaxel alone was 4.54 ± 1 nM which decreased to 3.03 ± 0.7, 1.9 ± 0.6 and 3.5 ± 0.8 nM upon combination with BT at 1.56, 6.25 and 25 μM, respectively (Fig 1D). These results show that when combined, BT and paclitaxel display synergy that depends on the concentration and sequence of addition of each drug.

#### A2780 (cisplatin-sensitive) and A2780-CDDP (cisplatin-resistant) isogenic pair

When A2780 cells were pretreated with BT followed by paclitaxel, drugs were mostly antagonistic except at high BT and paclitaxel concentrations, where synergism was observed. However, this synergism was non-significant as most of the cells were dead at such high concentrations of BT and paclitaxel, even when added individually. In contrast, pretreatment with paclitaxel followed by BT resulted in synergism only at concentrations ranging from 1.56 μM to 25 μM for BT and 0.98 to 7.81 nM for paclitaxel. On the other hand, simultaneous addition of BT and paclitaxel was synergistic at most of the drugs ratios (Fig 2A). After 48 hours of treatment, the BT IC_50_ value was 26 ± 4 μM while the IC_50_ value for paclitaxel alone was 7.2 ± 2 nM, which decreased to 5.9 ± 1, 4.6 ± 1, 3.5 ± 0.4, 5.5 ± 1 and 5.8 ± 0.8 nM upon combination with 1.56, 3.25, 6.25, 12.5 and 25 μM of BT, respectively (Fig 2B).

In A2780-CDDP (cisplatin-resistant) cells, additive effects were observed at specific drug ratios when cells were pretreated with BT followed by paclitaxel. Pretreatment with paclitaxel followed by BT resulted in synergism only at concentrations ranging from 1.56 to 25 μM for BT and 0.98 to 7.81 nM for paclitaxel (similarly to the cisplatin-sensitive variant of this isogenic pair). In contrast, simultaneous addition of BT and paclitaxel was synergistic at all drugs ratios (Fig 2C). After 48 hours of treatment, the IC_50_ value for BT was 25 ± 2 μM while the IC_50_ value for paclitaxel alone was 6 ± 1nM which decreased to 4.7 ± 1, 3.47 ± 0.5, 2.87 ± 0.3, 3.27 ± 1 and 3.46 ± 0.7 nM upon combination with 1.56, 3.25, 6.25 12.5 and 25 μM of BT, respectively (Fig 2D).

#### IGROV-1 (cisplatin-sensitive) and IGROV-1-CDDP (cisplatin-resistant) isogenic pair

Pretreatment of IGROV-1 cells with BT followed by paclitaxel addition, resulted in antagonism at almost all concentrations of both drugs. In contrast, pretreatment with paclitaxel followed by BT resulted in synergism at BT concentrations between 6.25 and 50 μM and paclitaxel concentrations between 0.98 and 7.81 nM. Simultaneous addition of both drugs resulted in synergism at most BT and paclitaxel concentrations (Fig 3A). After 48 hours of treatment, the IC_50_ value for BT was 87 ± 11 μM, whereas the IC_50_ value for paclitaxel alone was 13.2 ± 1 nM, which decreased to 4.7 ± 0.7, 3.7 ± 0.5, 2.5 ± 0.3, 3.5 ± 0.2 and 5.9 ± 0.7 nM upon combination with 1.56, 3.25, 6.25, 25, 50 μM BT, respectively (Fig 3B).

Similarly to IGROV-1, when IGROV-1-CDDP cells were pretreated with BT followed by paclitaxel, antagonism was observed at all concentrations of BT and paclitaxel. However, pretreatment with paclitaxel followed by BT resulted in synergism at the concentration ranges of 1.56 μM—50 μM for BT and 0.98 nM—7.81 nM for paclitaxel. Antagonism was observed at high concentrations of BT and paclitaxel. Similarly, simultaneous addition of BT and cisplatin was synergistic at most concentrations of both drugs (Fig 3C). After 48 hours of treatment, the IC_50_ value for BT was 94 ± 16 μM whereas the IC_50_ value for paclitaxel alone was 9.8 ± 1.2 nM, which decreased to 4.32 ± 0.2, 4.26 ± 0.4, 3.47 ± 0.3, 4.87 ± 0.8 and 5.79 ± 1 nM upon combination with 1.56, 3.25, 6.25, 25, 50 μM of BT, respectively (Fig 3D).

Overall, our results with these ovarian cancer cell lines show that the nature of BT-paclitaxel interaction is highly dependent on the sequence of addition and on the concentrations of both drugs. BT was synergistic to paclitaxel only when added simultaneously or when cells were pretreated with paclitaxel followed by BT. BT pretreatment desensitized the cells to paclitaxel treatment. The response to BT and paclitaxel combination was similar in all cell lines, regardless of their sensitivity to cisplatin.

#### BT enhances apoptosis when used in combination with paclitaxel

**Fig 4:**
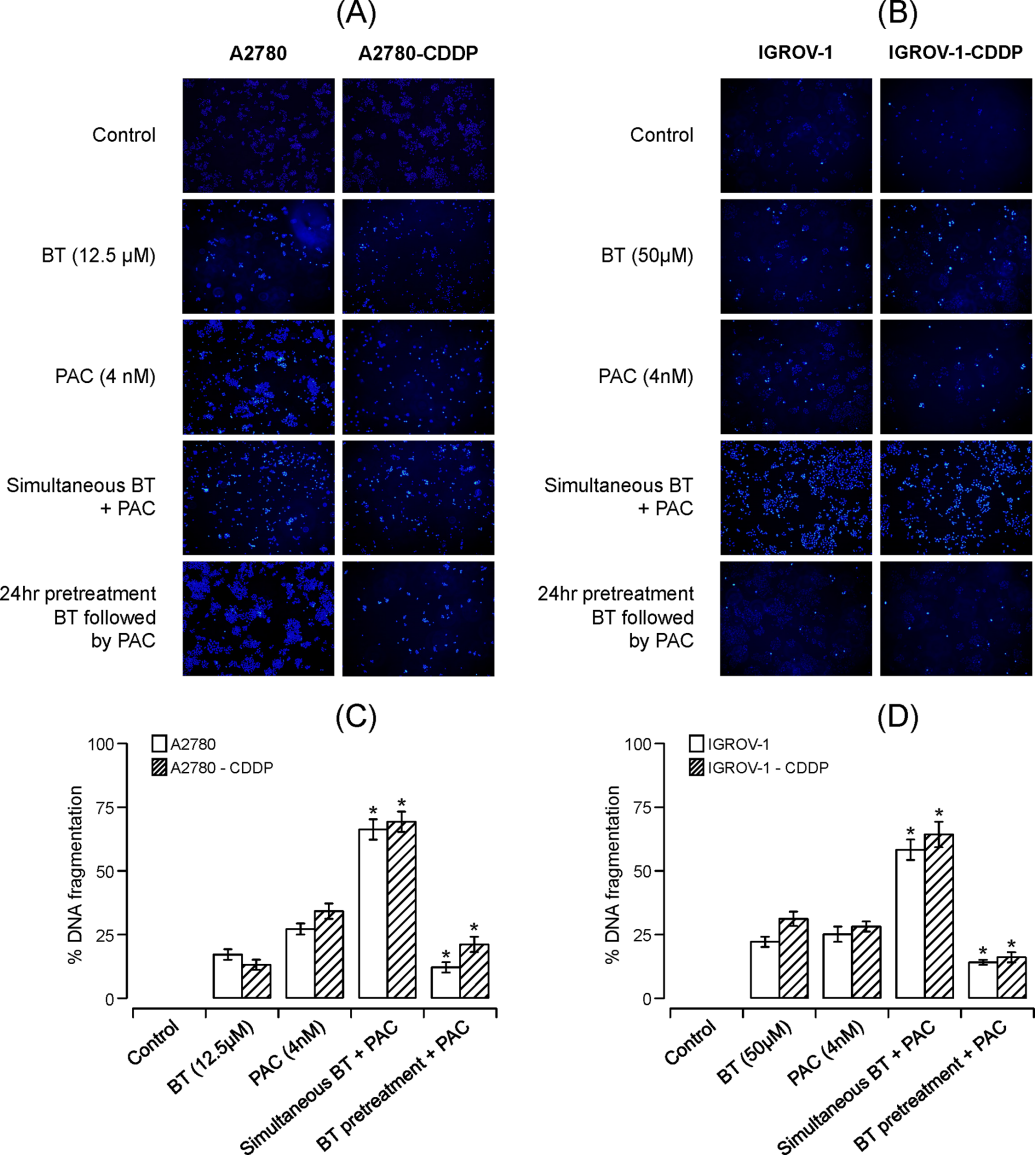
Apoptotic effects of BT-paclitaxel combination on isogenic pairs of ovarian cancer cell lines. **(A and C)** Representative images of Hoechst 33258 staining (for morphological, qualitative assessment of apoptosis) of A2780 and A2780-CDDP **(A)** or IGROV and IGROV-1-CDDP (**C)** cells treated with BT and/or paclitaxel as indicated. Percent of apoptosis in terms of DNA fragmentation (quantified via TUNEL assay) for A2780 and A2780-CDDP **(B)** or IGROV and 1/IGROV-1-CDDP **(D)** cells treated with BT or paclitaxel alone or in combination. The fragmentation observed in vehicle treated cells (control) is considered as zero percent against which treated cells were compared. Experiments were performed in duplicate. Data were expressed as means ± SD of duplicate experiments. Comparisons between paclitaxel alone treated and combination treated for each cell line were performed using Student’s t-test. Asterisks (*) indicate p < 0.05.

To understand the mechanism(s) underlying BT-paclitaxel synergism or antagonism, we evaluated the effect of BT on paclitaxel-induced apoptosis in cisplatin-sensitive and cisplatin-resistant isogenic pairs of cell lines such as A2780/A2780-CDDP and IGROV-1/IGROV-1-CDDP. Qualitative assessment of apoptosis was performed by nuclear (Hoechst) staining. Quantitative assessment of DNA fragmentation indicative of apoptosis was performed by TUNEL assay. As shown in Fig 4A and 4B, vehicle-treated (control) cells stained very faintly while drug-treated cells showed a stronger blue fluorescence. Stronger florescence implicates apoptosis due to highly condensed chromatin. All cell lines treated with BT-paclitaxel (added simultaneously) exhibited higher fluorescence than those treated with either drug alone. In contrast, cells pretreated with BT followed by paclitaxel exhibited lower fluorescence than the same cell lines treated with a single drug. Both isogenic pairs of cell lines showed similar trends. We confirmed these results by performing a TUNEL assay which also quantitates the extent of DNA fragmentation. The fragmentation observed in vehicle treated cells (control) is considered as zero percent against which treated cells were comparted. Consistent with the morphological assessment, the extent of apoptosis assayed by TUNEL (expressed as percentage of DNA fragmentation) was significantly higher when cells were simultaneously exposed to BT-paclitaxel versus treatment with either drug alone. Conversely, pretreatment with BT followed by paclitaxel reduced the percent of DNA fragmentation. This trend was observed in both isogenic cell pairs, regardless of their sensitivity to cisplatin (Fig 4C and 4D).

#### Effect of BT- paclitaxel combination on apoptotic markers

**Fig 5:**
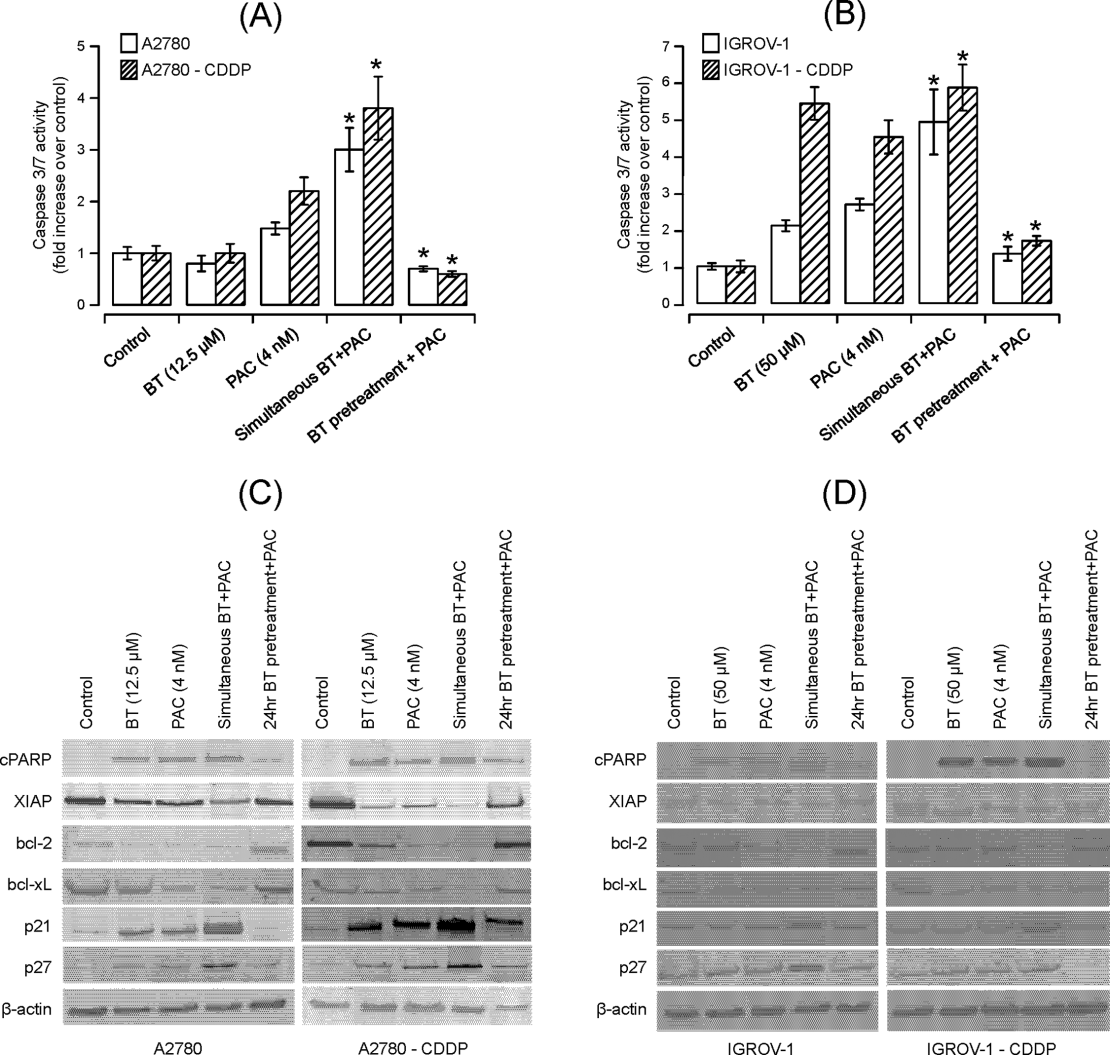
Assessment of apoptosis induced by BT-paclitaxel combination on ovarian cancer cell lines. The effect of BT-paclitaxel combination on caspase 3/7 activity was measured in **(A)** A2780 and A2780-CDDP or **(B)** IGROV-1 and IGROV-1-CDDP. Vehicle-treated cells were considered as control against which treated cells were compared. Data were expressed as means ± SD of triplicate experiments. Comparisons between paclitaxel alone-treated and combination-treated for each cell line were performed using Student’s t-test. Asterisks (*) indicate p < 0.05. The effects of BT-paclitaxel combination on pro-apoptotic (cPARP), anti-apoptotic (XIAP, bcl-2, bcl-xL) and cell cycle regulatory markers were assessed on A2780 and A2780-CDDP (**C)** or IGROV-1 and IGROV-1-CDDP **(D)**. Analysis of the expression of proteins in the lysates of treated and untreated (control) cells was carried out by SDS-PAGE and western blot analysis. The blots were probed with the respective primary antibodies. As an internal standard for equal loading, blots were probed with an anti-*β*-actin antibody. All blots were quantified using Odyssey software and data presented in the supporting information section, S2 Fig.

In order to complement the DNA fragmentation results, we also assessed other important apoptotic markers. A significant increase in caspases activity was observed when isogenic cell line pairs were treated with simultaneous addition of BT and paclitaxel, as compared to when treated with either of the drugs alone (Fig 5A and 5B). In contrast, pretreatment with BT followed by paclitaxel reduced caspase 3/7 activity as compared to treatment with either of the drugs alone (Fig 5A and 5B).

Although BT or paclitaxel alone increased the expression of PARP protein cleavage product (85 kDa, 1 fragment) in both isogenic cell pairs, simultaneous treatment with both agents caused a further increase (Fig 5C and 5D) in cPARP expression. In contrast, pretreatment with BT followed by paclitaxel reduced PARP protein cleavage compared to treatment with either of BT or paclitaxel alone.

To further confirm the contribution of apoptotic modulators in the BT enhancement of sensitivity to paclitaxel, we also assessed the expression of XIAP, bcl-2 and bcl-xL. As shown in Fig 5C and 5D, down regulation of XIAP, Bcl-2, bcl-xL was observed in both isogenic cell line pairs when treated with either drug alone. Treating cells with the BT-paclitaxel combination (simultaneous addition of drugs) further reduced expression of the aforementioned proteins compared to either agent alone. However, pretreating cells with BT followed by paclitaxel reduced paclitaxel-induced apoptotic effects as the expressions of XIAP, bcl-2 and bcl-xL increased compared to either agent alone.

#### Effect of BT-paclitaxel combination on key regulators of cell cycle

To understand the role of cell cycle regulators on the nature of the interactions (antagonism vs. synergism) between BT and paclitaxel, we assessed the expression of p27 (kip1) and p21. Our results show that BT and paclitaxel both enhanced expression of p27 *(kip1)* and p21 when used alone; however the extent of increase varied between the cell line pairs (Fig 5C and 5D). In the A2780/A2780-CDDP pair, an approximately 2–2.8 fold increase was observed while in the IGROV/IGROV1-CDDP pair the increase was slightly less (approximately 1.2–1.4 fold). In comparison, BT-paclitaxel combination further increased expression of p27 and p21 when these drugs were added simultaneously or decreased p27 and p21 when cells were pretreated with BT followed by paclitaxel. These results are consistent with our cytotoxicity data, where combination treatment (simultaneous addition) potentiated paclitaxel-induced cytotoxicity.

#### BT potentiates paclitaxel-induced apoptosis by increasing ROS generation in isogenic cancer cell line pairs

**Fig 6:**
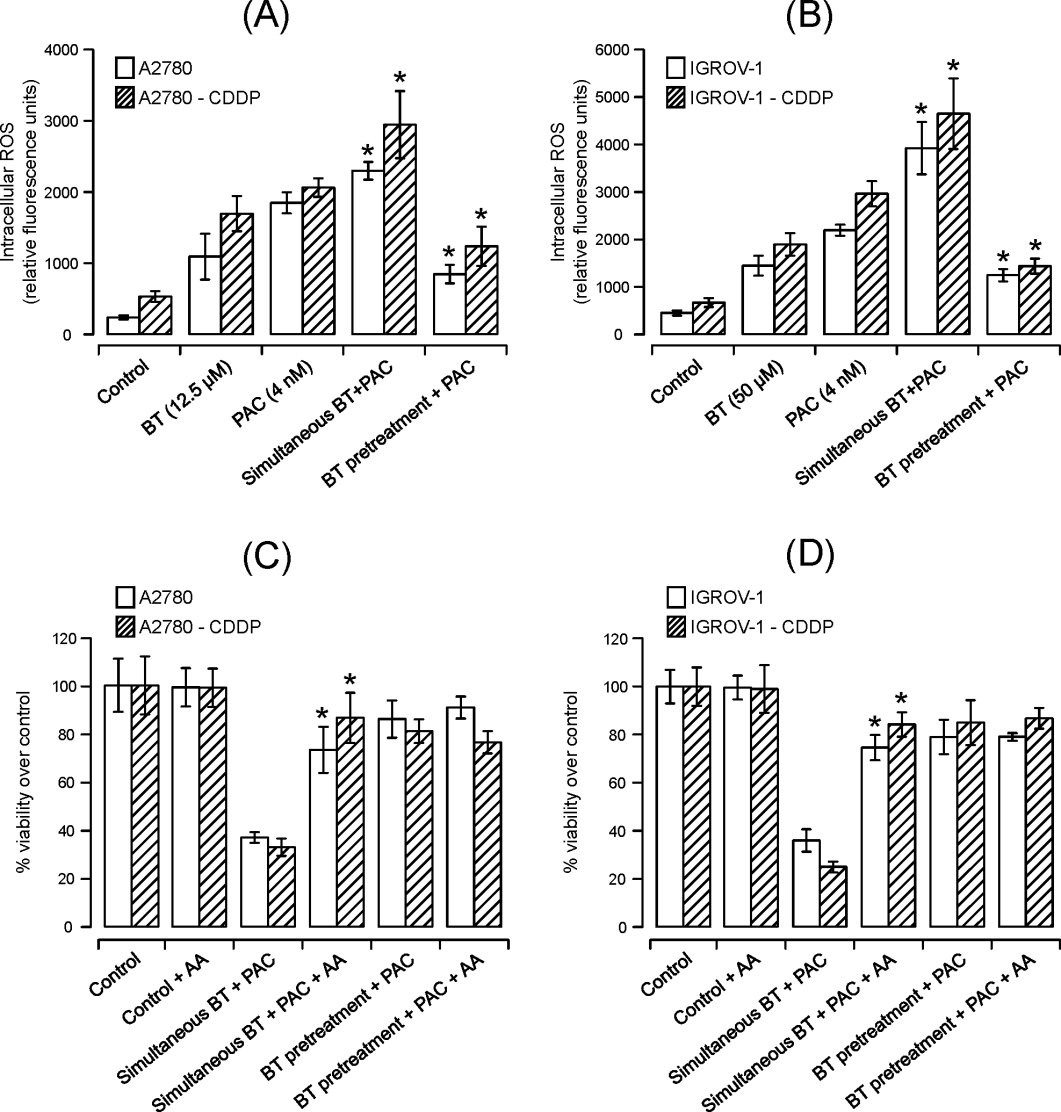
Assessment of intracellular ROS and antioxidant effect on ovarian cancer cells treated with BT-paclitaxel combination. Flow cytometry detection of intracellular ROS in A2780 and A2780-CDDP **(A)** or IGROV-1 and IGROV-1-CDDP **(B)** cells treated with BT or paclitaxel alone or in combination. Data were expressed as mean ± SD of triplicate experiments. Comparisons between paclitaxel alone treated and combination treated for each cell line were performed using Student’s t-test. Asterisks (*) indicate p < 0.05. **(C)** and **(D)** show the effect of the antioxidant ascorbic acid on the viability (via PrestoBlue) of A2780 and A2780-CDDP (**C)** or IGROV-1 and IGROV-1-CDDP **(D)** cells treated with BT or paclitaxel alone or in combination. Control (untreated) cells were considered as 100% viable against which treated cells were compared. Data were expressed as means ± SD of triplicate experiments. Comparisons between BT-paclitaxel-treated in presence of AA vs. combination-treated in the absence of AA for each cell line were performed using Student’s t-test. Asterisks (*) indicate p < 0.05.

We assessed ROS levels in order to understand the contribution of ROS towards the synergistic/antagonistic interaction between paclitaxel and BT. Consistent with our previous studies with BT [18], treatment with BT alone lead to an increase in ROS generation evidenced by an increase in fluorescence units (Fig 6A and 6B). Similarly, paclitaxel treatment also increased ROS generation. Compared to BT, paclitaxel caused greater generation of ROS in most of the cell lines (Fig 6A and 6B). Simultaneous treatment with paclitaxel and BT generated more ROS relative to either drug alone in all cell lines whereas pretreatment with BT followed by paclitaxel decreased ROS generation. To further confirm the role of ROS generation, we tested cell viability in the presence or absence of the antioxidant ascorbic acid (AA). As shown in Fig 6C and 6D, 1mM AA restored viability in cells treated simultaneously with BT and paclitaxel, e.g., 36% and 39% viability in cisplatin-sensitive cell lines (A2780 and IGROV-1) and 54% and 59% viability in cisplatin-resistant cell lines (A2780-CDDP and IGROV-1-CDDP), respectively. Interestingly, greater restoration of cell viability was observed in the cisplatin-resistant variants compared to cisplatin-sensitive cell lines. No significant restoration of viability by AA was observed when cells were pretreated with BT followed by paclitaxel. Treatment with 1 mM AA alone did not cause any loss in cell viability in any of the cell lines.

#### Effect of BT-cisplatin combination on ATX

**Fig 7:**
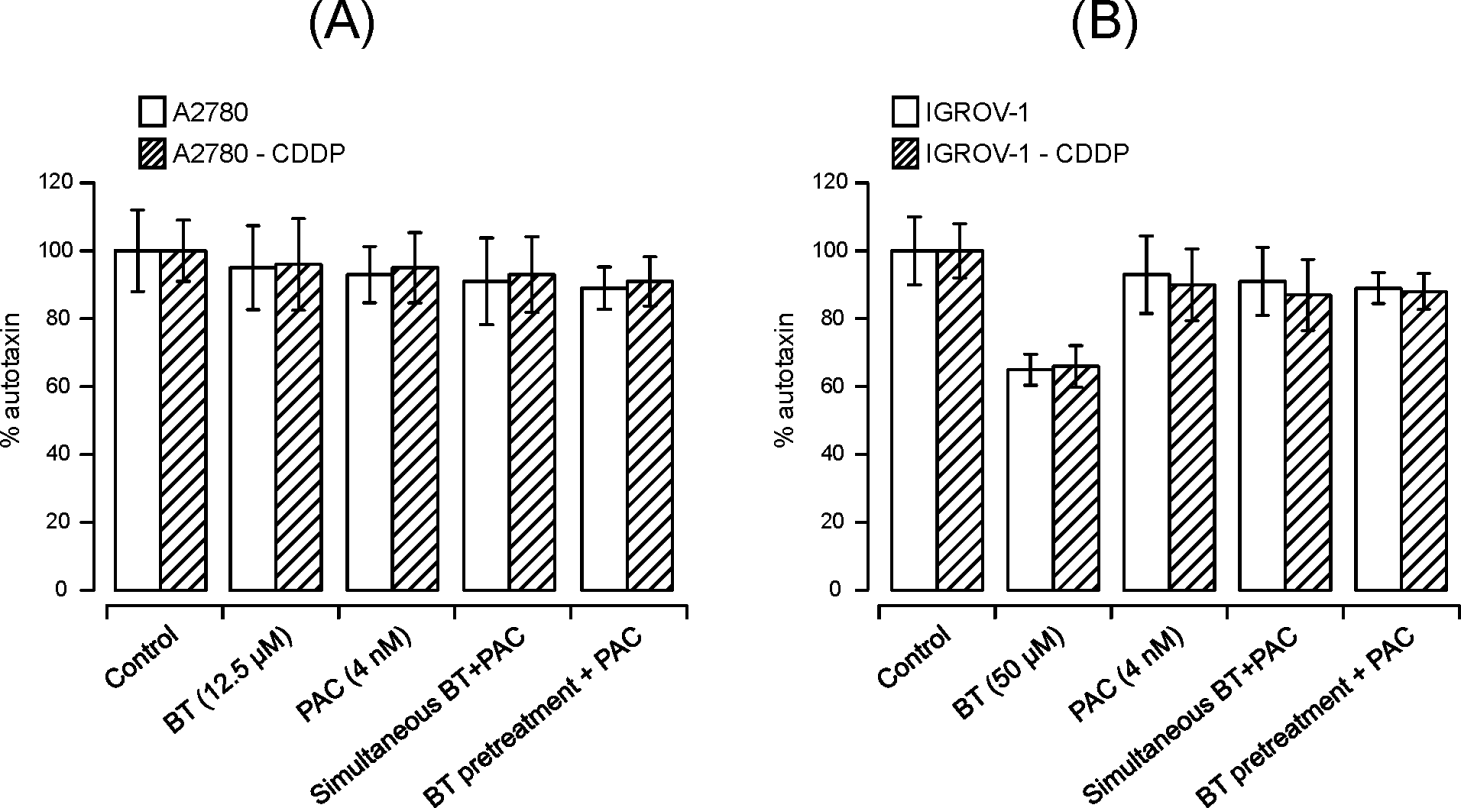
Effect of BT-paclitaxel combination on ATX secretion in ovarian cancer cell lines. A2780 and A2780-CDDP **(A)** or IGROV-1 and IGROV-1-CDDP **(B)** cells were treated with drugs either alone or in combination and ATX was measured from culture media (via a colorimetric assay). The percent of ATX inhibition in untreated cells was considered as reference against which treated cells were compared. Data were expressed as means ± SD of triplicate experiments. Comparisons between paclitaxel alone-treated and combination-treated for each cell line were performed using Student’s t-test. Asterisks (*) indicate p < 0.05.

ATX inhibition was considered the major mechanism of action for BT. To understand the contribution of ATX on the observed synergy between BT and paclitaxel, we determined the ATX levels in cell lysates treated with either drug alone or in combination (simultaneous addition). As shown in Fig 7A, treatment with 12.5 μM BT and 4 nM paclitaxel either by themselves or in combination did not affect ATX levels in A2780 or A2780-CDDP cells. Fig 7B shows that 4 nM paclitaxel did not cause significant changes in ATX levels in either IGROV-1 or IGROV1-CDDP cell lines (93 ± 3% and 90 ± 5%, respectively). In contrast, treatment with 50 μM BT caused a significant decrease in ATX levels in both cell lines (65% and 66%, respectively). However, when BT and paclitaxel were added in combination, ATX levels were not significantly different from those observed with paclitaxel alone.

## Discussion

Single-agent cancer therapy can rarely provide a satisfactory therapeutic effect in ovarian cancer patients due to drug resistance and dose-limiting side effects [21]. Combination chemotherapy has proven to be effective when each drug is administered at their maximum tolerated dose (MTD) to achieve maximum therapeutic efficiency. However, clinical data suggest that combination therapy is also unsuccessful because of drug resistance and side effects. One major reason attributed to this failure is exposure of tumor cells to drugs at antagonistic ratios or concentrations. Therefore, dosing at synergistic ratios is considered essential for successful combination therapy. Because anticancer drug combinations can act synergistically or antagonistically against tumor cells *in vitro* depending on the concentrations of the individual drugs, it is essential to determine optimal drug concentrations/ratios for every new drug combination to obtain synergy and to prevent the exposure of cells to antagonistic ratios.

Paclitaxel and cisplatin derivatives (e.g., carboplatin) are common chemotherapeutic agents used in the clinic for adjuvant treatment of ovarian cancer. The combination of these two drugs has been used following primary debulking surgery for ovarian cancer [22]. However, the major drawback of these combinations has been dose-limiting toxicities, therefore, it remains essential to discover novel compounds/chemosensitizers/modulators that enhance the therapeutic efficacy of paclitaxel or cisplatin, thus allowing their use at lower doses thereby reducing their toxic side effects. In previous studies, we showed that BT exerts cytotoxic effects by inducing apoptosis both *in vitro* and *in vivo* [16–18]. Our previous *in vitro* studies suggested that BT-induced cytotoxicity is due to increased ROS generation, NFkB inhibition and ATX inhibition. Enhanced cytotoxicity observed in cisplatin-resistant cell lines [16] was attributed to enhanced ROS generation in comparison to their cisplatin-sensitive isogenic partners. Additionally, we showed that pharmaceutical grade BT was well tolerated *in vivo* at relatively high concentrations without any toxic side effects although an anti-tumor effect was not observed at the doses tested [17]. The ability to induce apoptosis and lack of toxicity make BT a worthy candidate to be used in combination with cisplatin or paclitaxel in the treatment of ovarian cancer. In clinical scenarios, paclitaxel has been shown to be an effective agent in patients with relapsed platinum-refractory disease and is the most commonly used drug in “platinum resistance” settings [23, 24]. Furthermore, it has been suggested that the majority of cancer patients benefit from alternating therapy between cisplatin and paclitaxel [25]. After reporting a comprehensive study testing BT-cisplatin combination using a panel of ovarian cancer cell lines exhibiting varying sensitivities to cisplatin [18], we decided to further investigate BT’s potential to be used in combination with paclitaxel as a second-line (and/or beyond) therapy for treatment of recurrent ovarian cancer. In order to reflect “platinum-resistant” disease in ovarian cancer patients, we used the isogenic ovarian cancer cell lines pairs [A2780 (cisplatin-sensitive) / A2780-CDDP (cisplatin-resistant) and IGROV-1 (cisplatin-sensitive) / IGROV-1CDDP (cisplatin-resistant)] to understand the effectiveness of BT-paclitaxel combination and explore the mechanisms underlying the effects.

As reported by us and many others, drug combinations exhibit synergy at certain ratios and antagonism at other ratios. In our previous study, we reported that BT and cisplatin can be synergistic or antagonistic depending on the drugs concentrations/ratios. When used at optimal concentrations, BT potentiated cisplatin cytotoxicity, especially in cisplatin-resistant cell lines [18]. In this study, BT and paclitaxel combinations were evaluated systematically for concentration-dependent and sequence-dependent interactions *in vitro* using various treatment approaches. We found highly synergistic interactions between BT-paclitaxel when added simultaneously or when cells were pretreated with paclitaxel followed by BT. When added simultaneously, a synergistic interaction was observed over wide ranges of concentrations of BT and paclitaxel. However, when cells were pretreated with paclitaxel followed by BT, synergy was observed only at specific concentrations of BT and paclitaxel. In contrast, an antagonistic interaction was observed when cells were pretreated with BT followed by paclitaxel implying that BT desensitizes cells to paclitaxel response. Interestingly, all cell lines showed a similar response to the BT-paclitaxel combination, including both cisplatin-sensitive and cisplatin-resistant cell lines.

The mechanism(s) of action of chemotherapy drugs that induce cancer cell death by targeting DNA typically involves induction of apoptotic signaling and generation of oxidative stress [26, 27]. In the present study, we evaluated apoptosis, ROS generation, and expression of key apoptotic and cell cycle modulators as possible mechanisms of action for BT-paclitaxel combination. In our previous *in vitro* study, ATX inhibition was considered as one of the mechanism(s) of BT cytotoxic action [16, 28, 29]. In order to understand the contribution of ATX inhibition in BT-paclitaxel combination mediated cytotoxicity, we assessed the autotaxin activity in this *in vitro* study.

The majority of drug-induced apoptosis is attributed to disruption of mitochondrial potential resulting in release of cytochrome c into the cytosol and subsequent activation of caspases −3 and −7 which are executors of final steps of apoptosis [30–32]. It is well known that the apoptotic effect of paclitaxel is dependent on the concentration and exposure time. At concentrations less than 9 nM, paclitaxel-induced apoptosis is reported to be under the control Raf-1 whereas at concentrations greater than 9 nM, apoptosis occurs under the influence of p53 and p21 [33, 34]. Prolonged exposure to paclitaxel is known to cause irreversible mitotic arrest in addition to apoptosis. Additionally, low doses of paclitaxel induce immunomodulatory effects involving cytokines and pro-inflammatory proteins while higher doses cause cell death [35, 36]. Similarly, BT exhibited concertation dependent cytotoxicity with expression of pro-apoptotic markers and inhibition of anti-apoptotic/pro-survival markers at lower concentrations. When added simultaneously, the BT-paclitaxel combination displayed higher degrees of apoptosis and apoptotic markers (activation of caspase3/7, cleavage of PARP) in both cisplatin-resistant and cisplatin-sensitive cell line variants. However, when pretreated with paclitaxel followed by BT, synergy was observed only at specific drug ratios of BT and paclitaxel. Synergistic drug ratios of BT-paclitaxel combination may enhance the expression of pro-apoptotic signals resulting in enhanced cytotoxicity whereas antagonistic drug ratios may activate anti-apoptotic signals leading to drug resistance. These results indicate that combination chemotherapy is mechanistically unbiased due to the interplay of multiple mechanisms underlying the observed effects. When cells were pretreated with BT followed by paclitaxel, a decrease in apoptosis and apoptotic markers was observed.

Bad and Bax (apoptosis promoters) and bcl-2 (apoptosis suppressor) are bcl-2 family regulatory proteins involved in the programmed cell death [37]. It has been reported that fresh ovarian tissue biopsies contain higher levels of Bcl-2 along with p53 [38]. Expression of bcl-2 is important in protection from drug-induced apoptosis in ovarian cancer thereby contributing to chemo-resistance [38, 39]. Inhibition of bcl-xL may increase sensitivity to drugs such as carboplatin [40]. Paclitaxel is known to induce apoptosis via the mitogen-activated protein kinase (MAPK) pathway, resulting in dephosphorylation of pro-apoptotic proteins Bad and Bax and phosphorylation of bcl-2 [41]. BT is known to reduce bcl-2 in ovarian cancer cell lines resulting in apoptosis [16]. In this study, simultaneous treatment with BT-paclitaxel decreases the expressions of XIAP, bcl-2 and bcl-xL while pretreatment with BT followed by paclitaxel results in significant increased expression. These results correlate with the cytotoxicity data where simultaneous addition of BT-paclitaxel enhanced the cytotoxicity paclitaxel whereas pretreatment with BT followed by paclitaxel decreased it. Increased expression of XIAP and bcl-2 may be responsible for the lack of response when cells were pretreated with BT followed by paclitaxel. Our results also indicate that synergistic interactions between BT and paclitaxel may enhance apoptosis whereas antagonistic interactions lead to a reduction in apoptosis. These observations imply that the BT-paclitaxel combination may be a useful approach to treating ovarian cancer.

ROS generation is a key mechanism of apoptosis for a variety of common chemotherapeutic drugs such as daunorubicin, cyclophosphamide, cisplatin, and paclitaxel [42–45]. Many studies have shown that basal ROS levels in cancer cells are elevated relative to normal cells, which makes cancer cells more susceptible to oxidative damage due to further ROS increase upon treatment [46]. Paclitaxel had also been reported to induce ROS generation and increase hydroperoxide production by enhancing the activity of nicotinamide adenine dinucleotide phosphate (NADPH) oxidase, which contributes to oxidative stress and may play a role in paclitaxel’s anticancer properties [47, 48]. Generation of ROS has also been reported as one of the major mechanisms of BT induced cytotoxic activity [16, 49, 50]. Consistent with these previous reports, our current study shows that treatment with either BT or paclitaxel generated ROS. The combination of paclitaxel with BT further enhanced ROS generation but only when drugs were added simultaneously. Attenuation of ROS production by antioxidant AA protected cells from BT-paclitaxel induced cell death suggesting that ROS production is the major mechanism of cell death for the BT-paclitaxel combination. However, pretreatment with BT followed by paclitaxel, reduced ROS generation relative to treatment with either drug alone, suggesting possible desensitization to paclitaxel after continuous generation of ROS by BT pretreatment. Therefore enhanced elevation of cellular ROS level by simultaneous addition of both drugs has been proposed as a mechanism for targeting cancer cells.

Paclitaxel prevents cell division by promoting the assembly of stable microtubules and inhibiting their depolymerization, leading to cell arrest in G2/M-phase of the cell cycle [51] and eventually apoptosis [52]. These events are under the regulation of p53 and p21 [53, 54]. Previously, we reported that BT increases the expression of proteins P27 *(kip1)* and p21 resulting in cell cycle arrest at the G1/S phase in ovarian cancer cell lines *in vitro* [16]. In view of these results, we assessed the expression of key inhibitors of cell cycle P27 *(kip1)* and p21 in order to understand the role of these cell cycle regulators in causing antagonism or synergy in BT-paclitaxel treated cells. Our results suggest that when BT and paclitaxel are added simultaneously, BT enhances paclitaxel-induced cell cycle arrest by further increasing the expression of P27 *(kip1)* and p21 in all ovarian cancer cell lines. However, pretreatment with BT followed by paclitaxel, reduced expression of P27 *(kip1)* and p21 resulting in lower cytotoxicity of paclitaxel (antagonism). This suggests that cell cycle arrest at the G1/M phase by pretreatment with BT may have suppressed the cytotoxic effects of paclitaxel in mitotic arrest and apoptosis. Our results are consistent with the cytotoxicity data where BT-paclitaxel combination is highly schedule dependent.

BT was previously shown to inhibit tumor growth in several preclinical cancer models by targeting ATX [18, 28, 29]. ATX is known to increase the aggressiveness and invasiveness of transformed cells, and directly correlates with tumor stage and grade in several human malignancies, including ovarian cancer [55, 56]. In this study, we assessed ATX levels in order to understand the contribution of BT mediated ATX inhibition to the cytotoxic potential of BT-paclitaxel combination treatments. We did not find any significant difference when any of the isogenic pairs were treated with BT-paclitaxel combination. These results suggest that ATX inhibition does not play a critical role in the mechanism of action of the BT-paclitaxel combination.

BT is well tolerated in humans. An early report showed that 50 mg/kg in three divided alternate daily doses for 5 days will maintain serum levels of BT in the range of 140 to 550 μM in rabbits, dogs and humans [15]. However, a more recent study reported that in a patient with refractory *C*. *neoformans* meningitis, dosing with bithionol at 50 mg/kg/day for 3 days resulted in trough plasma drug levels of only 36.3, 46.3, and 44.9 μg/ml on days 1, 2, and 3, respectively, with a simultaneous drug level of 46.2 μg/ml in the plasma [57]. Although observed BT IC_50_ values and the proposed BT concentrations to be used in combination with paclitaxel are below the reported clinical values, there is still a need to further improve the bioavailability by various strategies. In a previous *in vivo* study we reported that pharma grade BT did not exhibit significant anti-tumor potential at any of the doses when tested using a mouse xenograft model of epithelial ovarian cancer [17]. Pharma grade BT was administered orally via gavage (the contents of BT capsules were suspended in water). The observed lack of therapeutic effect was attributed to poor water solubility and the disease stage at which we started the treatment. Other limitations in obtaining effective BT concentrations *in vivo* include protein binding and poor cellular diffusion [29]. Therefore, further optimization (including different formulations, vehicles, routes of delivery and wide dose ranges) is necessary to enhance the solubility and absorption of BT in order to administer effective doses either alone or in combination with other drugs. Increasing the bioavailability by eliminating chromatin-damaging phenolic groups may enhance the effectiveness of the agent [58].

Evaluation of drug interactions in vitro is a prerequisite for testing new drug combinations in vivo. As reported by many, drug ratios identified as antagonistic in vitro provide inferior therapeutic activity in vivo compared with a synergistic drug ratio. Additionally, combination therapy may yield significant clinical benefits only when effective drug concentrations can be attained in the body while maintaining the synergistic ratios. Translation of in vitro information on drug ratio-dependent synergy to in vivo can be done using various drug delivery technologies. Combination drug delivery systems such as polymeric nanoparticles, dendrimers, liposomes, and water-soluble polymer-drug conjugates can be used to obtain synergistic anticancer effects while reducing concentration-related toxicity of the individual drugs [59, 60].

In the present study, we evaluated the nature of BT-paclitaxel interactions *in vitro*. Our results show that in most ovarian cancer cell lines tested, paclitaxel and BT displayed synergy when combined at lower doses. The use of lower doses of paclitaxel would potentially diminish its toxic side effects without compromising efficacy. Although the anti-tumor potential of pharmaceutical grade BT was not observed in our *in vivo* study [17], the ability of BT to induce apoptosis, its lack of toxicity at any of the doses tested *in vivo* and the ability to interact synergistically with Paclitaxel and Cisplatin are important properties that merit further investigation.

## Conclusions

Our results show that BT enhances sensitivity of ovarian cancer cell lines to paclitaxel treatment at most drug ratios. Increased ROS generation and modulation of key regulators of apoptosis and the cell cycle may have contributed to this enhanced sensitivity. The nature of BT-paclitaxel interaction is highly dependent on the sequence of addition of drugs. Simultaneous addition or pretreatment with paclitaxel followed by BT results in synergistic interactions. Our results suggest that novel combinations such as BT and paclitaxel may provide a promising therapeutic strategy to enhance the chemosensitivity of ovarian cancer while minimizing side effects. *In vivo* experiments may aid in determining the role of BT (and BT combinations) in the treatment of patients with ovarian cancer.

## Supporting information

S1 Table IC_50_ values of BT and paclitaxel in various ovarian cancer cell lines, at 48 hours post-treatment. (PDF)

S1 Fig Cells were treated with bithionol at concentrations ranging from 0.178 μM to 400 μM to calculate the concentration of drugs required to achieve 50% growth inhibition (IC_50_). The experiments were repeated at least three times with different cellular passages. IC_50_ values are expressed as mean ± SD.
(PDF)

S2 Fig Western blot quantification of various apoptotic and cell cycle regulatory molecules presented in Fig 5C (A) and 5D (B). Data represent fold difference over control cells (vehicle treated only). Values are means±S.E.M. of three independent experiments. Asterisks (*) denote significant difference, at P < 0.05, as compared to cells treated with paclitaxel alone.
(PDF)

## Abbreviations

BT: Bithionol
ATX: Autotaxin
IC_50_: Half maximal inhibitory concentration
ROS: Reactive oxygen species
NF-κB: Nuclear factor kappa B
XIAP: x linked inhibitor of apoptosis, IGROV-1CDDP/ A2780- CDDP–cisplatin-resistant variants of IGROV-1 and A2780

## References

[1] Kikuchi Y. [The mechanism of cisplatin-resistance in ovarian cancer]. Hum Cell. 2001;14(2):115–33. . 11552292

[2] Leitao MM, Hummer A, Dizon DS, Aghajanian C, Hensley M, Sabbatini P, et al Platinum retreatment of platinum-resistant ovarian cancer after nonplatinum therapy. Gynecol Oncol. 2003;91(1):123–9. . 14529671 10.1016/s0090-8258(03)00464-5

[3] Lambert HE, Gregory WM, Nelstrop AE, Rustin GJ. Long-term survival in 463 women treated with platinum analogs for advanced epithelial carcinoma of the ovary: life expectancy compared to women of an age-matched normal population. Int J Gynecol Cancer. 2004;14(5):772–8. doi: 10.1111/j.1048-891X.2004.014507.x . 15361183 10.1111/j.1048-891X.2004.014507.x

[4] Ott I, Gust R. Non platinum metal complexes as anti-cancer drugs. Arch Pharm (Weinheim). 2007;340(3):117–26. doi: 10.1002/ardp.200600151 . 17315259 10.1002/ardp.200600151

[5] Rowinsky EK, Donehower RC. Paclitaxel (taxol). N Engl J Med. 1995;332(15):1004–14. Epub 1995/04/13. doi: 10.1056/NEJM199504133321507 . 7885406 10.1056/NEJM199504133321507

[6] Band Horwitz S. Mechanism of action of taxol. Trends in Pharmacological Sciences. 13:134–6. doi: 10.1016/0165-6147(92)90048-B 1350385 10.1016/0165-6147(92)90048-b

[7] Woods CM, Zhu J, McQueney PA, Bollag D, Lazarides E. Taxol-induced mitotic block triggers rapid onset of a p53-independent apoptotic pathway. Mol Med. 1995;1(5):506–26. Epub 1995/07/01. ; PubMed Central PMCID: PMCPMC2229961. 8529117 PMC2229961

[8] Sherman-Baust CA, Becker KG, Wood WH Iii, Zhang Y, Morin PJ. Gene expression and pathway analysis of ovarian cancer cells selected for resistance to cisplatin, paclitaxel, or doxorubicin. J Ovarian Res. 2011;4(1):21 Epub 2011/12/07. doi: 10.1186/1757-2215-4-21 ; PubMed Central PMCID: PMCPMC3259089. 22141344 10.1186/1757-2215-4-21 PMC3259089

[9] Agarwal R, Kaye SB. Ovarian cancer: strategies for overcoming resistance to chemotherapy. Nat Rev Cancer. 2003;3(7):502–16. doi: 10.1038/nrc1123 . 12835670 10.1038/nrc1123

[10] Lipton RB, Apfel SC, Dutcher JP, Rosenberg R, Kaplan J, Berger A, et al Taxol produces a predominantly sensory neuropathy. Neurology. 1989;39(3):368–73. Epub 1989/03/01. . 2564647 10.1212/wnl.39.3.368

[11] Rowinsky EK, Chaudhry V, Cornblath DR, Donehower RC. Neurotoxicity of Taxol. J Natl Cancer Inst Monogr. 1993;(15):107–15. Epub 1993/01/01. . 7912516

[12] Pace A, Bove L, Aloe A, Nardi M, Pietrangeli A, Calabresi F, et al Paclitaxel neurotoxicity: clinical and neurophysiological study of 23 patients. Ital J Neurol Sci. 1997;18(2):73–9. Epub 1997/04/01. . 9239526 10.1007/BF01999566

[13] Sui M, Xiong X, Kraft AS, Fan W. Combination of gemcitabine antagonizes antitumor activity of paclitaxel through prevention of mitotic arrest and apoptosis. Cancer Biol Ther. 2006;5(8):1015–21. Epub 2006/07/21. . 16855376 10.4161/cbt.5.8.2909

[14] Zeng S, Chen YZ, Fu L, Johnson KR, Fan W. In vitro evaluation of schedule-dependent interactions between docetaxel and doxorubicin against human breast and ovarian cancer cells. Clin Cancer Res. 2000;6(9):3766–73. Epub 2000/09/22. . 10999771

[15] Bacq Y, Besnier JM, Duong TH, Pavie G, Metman EH, Choutet P. Successful treatment of acute fascioliasis with bithionol. Hepatology. 1991;14(6):1066–9. . 1959855

[16] Ayyagari VN, Brard L. Bithionol inhibits ovarian cancer cell growth in vitro—studies on mechanism(s) of action. BMC Cancer. 2014;14:61 doi: 10.1186/1471-2407-14-61 ; PubMed Central PMCID: PMCPMC3922745. 24495391 10.1186/1471-2407-14-61 PMC3922745

[17] Ayyagari VN, Johnston NA, Brard L. Assessment of the antitumor potential of Bithionol in vivo using a xenograft model of ovarian cancer. Anticancer Drugs. 2016;27(6):547–59. doi: 10.1097/CAD.0000000000000364 . 27058706 10.1097/CAD.0000000000000364 PMC5053334

[18] Ayyagari VN, Hsieh TJ, Diaz-Sylvester PL, Brard L. Evaluation of the cytotoxicity of the Bithionol—cisplatin combination in a panel of human ovarian cancer cell lines. BMC Cancer. 2017;17(1):49 Epub 2017/01/15. doi: 10.1186/s12885-016-3034-2 ; PubMed Central PMCID: PMCPMC5234112. 28086831 10.1186/s12885-016-3034-2 PMC5234112

[19] Chou TC. Drug combination studies and their synergy quantification using the Chou-Talalay method. Cancer Res. 2010;70(2):440–6. doi: 10.1158/0008-5472.CAN-09-1947 . 20068163 10.1158/0008-5472.CAN-09-1947

[20] Kasibhatla S, Amarante-Mendes GP, Finucane D, Brunner T, Bossy-Wetzel E, Green DR. Staining of suspension cells with hoechst 33258 to detect apoptosis. CSH Protoc. 2006;2006(3). doi: 10.1101/pdb.prot4492 . 22485873 10.1101/pdb.prot4492

[21] Naumann RW, Coleman RL. Management strategies for recurrent platinum-resistant ovarian cancer. Drugs. 2011;71(11):1397–412. doi: 10.2165/11591720-000000000-00000 . 21812505 10.2165/11591720-000000000-00000

[22] Cristea M, Han E, Salmon L, Morgan RJ. Practical considerations in ovarian cancer chemotherapy. Ther Adv Med Oncol. 2010;2(3):175–87. Epub 2010/05/01. doi: 10.1177/1758834010361333 ; PubMed Central PMCID: PMCPMC3126016. 21789133 10.1177/1758834010361333 PMC3126016

[23] Piccart MJ, Green JA, Lacave AJ, Reed N, Vergote I, Benedetti-Panici P, et al Oxaliplatin or paclitaxel in patients with platinum-pretreated advanced ovarian cancer: A randomized phase II study of the European Organization for Research and Treatment of Cancer Gynecology Group. J Clin Oncol. 2000;18(6):1193–202. Epub 2000/03/15. doi: 10.1200/JCO.2000.18.6.1193 . 10715288 10.1200/JCO.2000.18.6.1193

[24] Pinato DJ, Graham J, Gabra H, Sharma R. Evolving concepts in the management of drug resistant ovarian cancer: dose dense chemotherapy and the reversal of clinical platinum resistance. Cancer Treat Rev. 2013;39(2):153–60. Epub 2012/05/19. doi: 10.1016/j.ctrv.2012.04.004 . 22595680 10.1016/j.ctrv.2012.04.004

[25] Rosenberg P, Andersson H, Boman K, Ridderheim M, Sorbe B, Puistola U, et al Randomized trial of single agent paclitaxel given weekly versus every three weeks and with peroral versus intravenous steroid premedication to patients with ovarian cancer previously treated with platinum. Acta Oncol. 2002;41(5):418–24. Epub 2002/11/22. . 12442916 10.1080/028418602320404998

[26] Hwang PM, Bunz F, Yu J, Rago C, Chan TA, Murphy MP, et al Ferredoxin reductase affects p53-dependent, 5-fluorouracil-induced apoptosis in colorectal cancer cells. Nat Med. 2001;7(10):1111–7. doi: 10.1038/nm1001-1111 ; PubMed Central PMCID: PMCPMC4086305. 11590433 10.1038/nm1001-1111 PMC4086305

[27] Kim KK, Kawar NM, Singh RK, Lange TS, Brard L, Moore RG. Tetrathiomolybdate induces doxorubicin sensitivity in resistant tumor cell lines. Gynecol Oncol. 2011;122(1):183–9. doi: 10.1016/j.ygyno.2011.03.035 . 21529906 10.1016/j.ygyno.2011.03.035

[28] Braddock D, inventorApplication WO2009151644 A2. Yale University, USA (2009). Small molecule inhibitors of autotaxin, and methods of use. This patent is notable as one of only two that include demonstration in vivo of anti- cancer activity of an ATX inhibitor.

[29] Saunders LP, Ouellette A, Bandle R, Chang WC, Zhou H, Misra RN, et al Identification of small-molecule inhibitors of autotaxin that inhibit melanoma cell migration and invasion. Mol Cancer Ther. 2008;7(10):3352–62. doi: 10.1158/1535-7163.MCT-08-0463 . 18852138 10.1158/1535-7163.MCT-08-0463 PMC7857123

[30] Zamzami N, Marchetti P, Castedo M, Decaudin D, Macho A, Hirsch T, et al Sequential reduction of mitochondrial transmembrane potential and generation of reactive oxygen species in early programmed cell death. J Exp Med. 1995;182(2):367–77. ; PubMed Central PMCID: PMCPMC2192111. 7629499 10.1084/jem.182.2.367 PMC2192111

[31] Kaufmann SH, Earnshaw WC. Induction of apoptosis by cancer chemotherapy. Exp Cell Res. 2000;256(1):42–9. doi: 10.1006/excr.2000.4838 . 10739650 10.1006/excr.2000.4838

[32] Kaufmann SH, Gores GJ. Apoptosis in cancer: cause and cure. Bioessays. 2000;22(11):1007–17. doi: 10.1002/1521-1878(200011)22:11<1007::AID-BIES7>3.0.CO;2-4 . 11056477 10.1002/1521-1878(200011)22:11<1007::AID-BIES7>3.0.CO;2-4

[33] Sevko A, Kremer V, Falk C, Umansky L, Shurin MR, Shurin GV, et al Application of paclitaxel in low non-cytotoxic doses supports vaccination with melanoma antigens in normal mice. J Immunotoxicol. 2012;9(3):275–81. Epub 2012/03/28. doi: 10.3109/1547691X.2012.655343 . 22449053 10.3109/1547691X.2012.655343

[34] Kreis T, Vale R. Guidebook to the Cytoskeletal and Motor Proteins. 2nd ed. Oxford, UK: Oxford University Press; 1999.

[35] Giannakakou P, Sackett DL, Kang YK, Zhan Z, Buters JT, Fojo T, et al Paclitaxel-resistant human ovarian cancer cells have mutant beta-tubulins that exhibit impaired paclitaxel-driven polymerization. J Biol Chem. 1997;272(27):17118–25. Epub 1997/07/04. . 9202030 10.1074/jbc.272.27.17118

[36] Rakovitch E, Mellado W, Hall EJ, Pandita TK, Sawant S, Geard CR. Paclitaxel sensitivity correlates with p53 status and DNA fragmentation, but not G2/M accumulation. Int J Radiat Oncol Biol Phys. 1999;44(5):1119–24. Epub 1999/07/27. . 10421546 10.1016/s0360-3016(99)00109-1

[37] Marx D, Meden H. Differential Expression of Apoptosis-Associated Genes bax and bcl-2 in Ovarian Cancer. Methods Mol Med. 2001;39:687–91. Epub 2001/01/01. doi: 10.1385/1-59259-071-3:687 . 21340831 10.1385/1-59259-071-3:687

[38] Eliopoulos AG, Kerr DJ, Herod J, Hodgkins L, Krajewski S, Reed JC, et al The control of apoptosis and drug resistance in ovarian cancer: influence of p53 and Bcl-2. Oncogene. 1995;11(7):1217–28. . 7478541

[39] Herod JJ, Eliopoulos AG, Warwick J, Niedobitek G, Young LS, Kerr DJ. The prognostic significance of Bcl-2 and p53 expression in ovarian carcinoma. Cancer Res. 1996;56(9):2178–84. . 8616869

[40] Witham J, Valenti MR, De-Haven-Brandon AK, Vidot S, Eccles SA, Kaye SB, et al The Bcl-2/Bcl-XL family inhibitor ABT-737 sensitizes ovarian cancer cells to carboplatin. Clin Cancer Res. 2007;13(23):7191–8. doi: 10.1158/1078-0432.CCR-07-0362 . 18056200 10.1158/1078-0432.CCR-07-0362

[41] Kampan NC, Madondo MT, McNally OM, Quinn M, Plebanski M. Paclitaxel and Its Evolving Role in the Management of Ovarian Cancer. Biomed Res Int. 2015;2015:413076 Epub 2015/07/03. doi: 10.1155/2015/413076 ; PubMed Central PMCID: PMCPMC4475536. 26137480 10.1155/2015/413076 PMC4475536

[42] Kim JS, Lee JH, Jeong WW, Choi DH, Cha HJ, Kim DH, et al Reactive oxygen species-dependent EndoG release mediates cisplatin-induced caspase-independent apoptosis in human head and neck squamous carcinoma cells. Int J Cancer. 2008;122(3):672–80. doi: 10.1002/ijc.23158 . 17955488 10.1002/ijc.23158

[43] Mansat-de Mas V, Bezombes C, Quillet-Mary A, Bettaïeb A, D'orgeix AD, Laurent G, et al Implication of radical oxygen species in ceramide generation, c-Jun N-terminal kinase activation and apoptosis induced by daunorubicin. Mol Pharmacol. 1999;56(5):867–74. . 10531389 10.1124/mol.56.5.867

[44] Tsai-Turton M, Luong BT, Tan Y, Luderer U. Cyclophosphamide-induced apoptosis in COV434 human granulosa cells involves oxidative stress and glutathione depletion. Toxicol Sci. 2007;98(1):216–30. doi: 10.1093/toxsci/kfm087 . 17434952 10.1093/toxsci/kfm087

[45] Schiff PB, Fant J, Horwitz SB. Promotion of microtubule assembly in vitro by taxol. Nature. 1979;277(5698):665–7. Epub 1979/02/22. . 423966 10.1038/277665a0

[46] Trachootham D, Alexandre J, Huang P. Targeting cancer cells by ROS-mediated mechanisms: a radical therapeutic approach? Nat Rev Drug Discov. 2009;8(7):579–91. doi: 10.1038/nrd2803 . 19478820 10.1038/nrd2803

[47] Alexandre J, Hu Y, Lu W, Pelicano H, Huang P. Novel action of paclitaxel against cancer cells: bystander effect mediated by reactive oxygen species. Cancer Res. 2007;67(8):3512–7. Epub 2007/04/19. doi: 10.1158/0008-5472.CAN-06-3914 . 17440056 10.1158/0008-5472.CAN-06-3914

[48] Hadzic T, Aykin-Burns N, Zhu Y, Coleman MC, Leick K, Jacobson GM, et al Paclitaxel combined with inhibitors of glucose and hydroperoxide metabolism enhances breast cancer cell killing via H2O2-mediated oxidative stress. Free Radic Biol Med. 2010;48(8):1024–33. Epub 2010/01/20. doi: 10.1016/j.freeradbiomed.2010.01.018 ; PubMed Central PMCID: PMCPMC2843822. 20083194 10.1016/j.freeradbiomed.2010.01.018 PMC2843822

[49] Meshkini A, Yazdanparast R. Involvement of oxidative stress in taxol-induced apoptosis in chronic myelogenous leukemia K562 cells. Exp Toxicol Pathol. 2012;64(4):357–65. doi: 10.1016/j.etp.2010.09.010 . 21074392 10.1016/j.etp.2010.09.010

[50] Berndtsson M, Hägg M, Panaretakis T, Havelka AM, Shoshan MC, Linder S. Acute apoptosis by cisplatin requires induction of reactive oxygen species but is not associated with damage to nuclear DNA. Int J Cancer. 2007;120(1):175–80. doi: 10.1002/ijc.22132 . 17044026 10.1002/ijc.22132

[51] Schiff PB, Horwitz SB. Taxol stabilizes microtubules in mouse fibroblast cells. Proc Natl Acad Sci U S A. 1980;77(3):1561–5. Epub 1980/03/01. ; PubMed Central PMCID: PMCPMC348536. 6103535 10.1073/pnas.77.3.1561 PMC348536

[52] Zhang D, Yang R, Wang S, Dong Z. Paclitaxel: new uses for an old drug. Drug Des Devel Ther. 2014;8:279–84. Epub 2014/03/05. doi: 10.2147/DDDT.S56801 ; PubMed Central PMCID: PMCPMC3934593. 24591817 10.2147/DDDT.S56801 PMC3934593

[53] Mitsuuchi Y, Johnson SW, Selvakumaran M, Williams SJ, Hamilton TC, Testa JR. The phosphatidylinositol 3-kinase/AKT signal transduction pathway plays a critical role in the expression of p21WAF1/CIP1/SDI1 induced by cisplatin and paclitaxel. Cancer Res. 2000;60(19):5390–4. . 11034077

[54] He G, Kuang J, Huang Z, Koomen J, Kobayashi R, Khokhar AR, et al Upregulation of p27 and its inhibition of CDK2/cyclin E activity following DNA damage by a novel platinum agent are dependent on the expression of p21. Br J Cancer. 2006;95(11):1514–24. doi: 10.1038/sj.bjc.6603448 ; PubMed Central PMCID: PMCPMC2360737. 17088910 10.1038/sj.bjc.6603448 PMC2360737

[55] Nam SW, Clair T, Campo CK, Lee HY, Liotta LA, Stracke ML. Autotaxin (ATX), a potent tumor motogen, augments invasive and metastatic potential of ras-transformed cells. Oncogene. 2000;19(2):241–7. doi: 10.1038/sj.onc.1203263 . 10645002 10.1038/sj.onc.1203263

[56] Nam SW, Clair T, Kim YS, McMarlin A, Schiffmann E, Liotta LA, et al Autotaxin (NPP-2), a metastasis-enhancing motogen, is an angiogenic factor. Cancer Res. 2001;61(18):6938–44. . 11559573

[57] Park YD, Sun W, Salas A, Antia A, Carvajal C, Wang A, et al Identification of Multiple Cryptococcal Fungicidal Drug Targets by Combined Gene Dosing and Drug Affinity Responsive Target Stability Screening. MBio. 2016;7(4). Epub 2016/08/04. doi: 10.1128/mBio.01073-16 ; PubMed Central PMCID: PMCPMC4981720. 27486194 10.1128/mBio.01073-16 PMC4981720

[58] Chen H, Eastmond DA. Synergistic increase in chromosomal breakage within the euchromatin induced by an interaction of the benzene metabolites phenol and hydroquinone in mice. Carcinogenesis. 1995;16(8):1963–9. Epub 1995/08/01. . 7543378 10.1093/carcin/16.8.1963

[59] Allen TM, Cullis PR. Drug delivery systems: entering the mainstream. Science. 2004;303(5665):1818–22. doi: 10.1126/science.1095833 . 15031496 10.1126/science.1095833

[60] Torchilin VP. Targeted pharmaceutical nanocarriers for cancer therapy and imaging. AAPS J. 2007;9(2):E128–47. doi: 10.1208/aapsj0902015 ; PubMed Central PMCID: PMCPMC2751402. 17614355 10.1208/aapsj0902015 PMC2751402

