# Supplementary material for: Evaluation of the cytotoxicity of the Bithionol-paclitaxel combination in a panel of human ovarian cancer cell lines
Source: PLoS One. 2017 Sep 20;12(9):e0185111. doi: 10.1371/journal.pone.0185111 (PMC5607185; doi:10.1371/journal.pone.0185111)
Supplement: S1 Table — (PDF) [file pone.0185111.s001.pdf]

S1 Table. IC_{50} Values of BT and Paclitaxel in various ovarian cancer cell lines, at 48 hours post-treatment.

	Bithionol [µM]	Paclitaxel [nM]
OVACAR-3	57 ± 8	5.05 ± 1
SKOV-3	58 ± 3	4.54 ± 1
IGROV-1	87 ± 11	13.2 ± 1
IGROV-1 CDDP	94 ± 16	9.81 ± 1
A2780	26 ± 4	7.20 ± 2
A2780-CDDP	25 ± 2	6.02 ± 2
