# Supplementary material for: Evaluation of the cytotoxicity of the Bithionol-paclitaxel combination in a panel of human ovarian cancer cell lines
Source: PLoS One. 2017 Sep 20;12(9):e0185111. doi: 10.1371/journal.pone.0185111 (PMC5607185; doi:10.1371/journal.pone.0185111)
Supplement: S1 Fig — The experiments were repeated at least three times with different cellular passages. IC50 values are expressed as mean ± SD. (PDF) [file pone.0185111.s002.pdf]

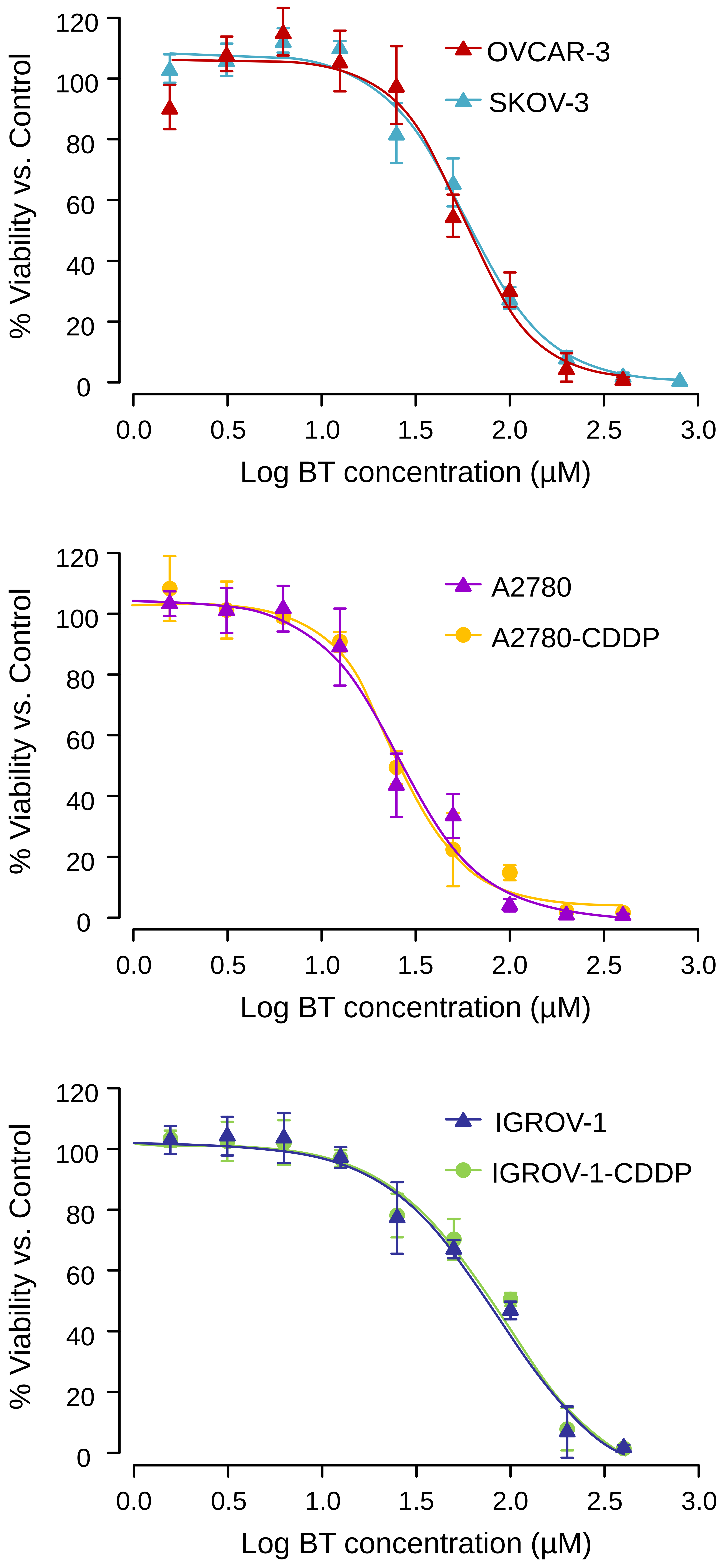

S1 Fig. Cells were treated with Bithionol at concentrations ranging from 0.178 µM to 400 µM to calculate the concentration of drugs required to achieve 50% growth inhibition (IC_{50}). The experiments were repeated at least three times with different cellular passages. IC_{50} values are expressed as mean ± SD.
