# Supplementary material for: Evaluation of the cytotoxicity of the Bithionol-paclitaxel combination in a panel of human ovarian cancer cell lines
Source: PLoS One. 2017 Sep 20;12(9):e0185111. doi: 10.1371/journal.pone.0185111 (PMC5607185; doi:10.1371/journal.pone.0185111)
Supplement: S2 Fig — Data represent fold difference over control cells (vehicle treated only). Values are means±S.E.M. of three independent experiments. Asterisks (*) denote significant difference, at P < 0.05, as compared to cells treated with paclitaxel alone. (PDF) [file pone.0185111.s003.pdf]

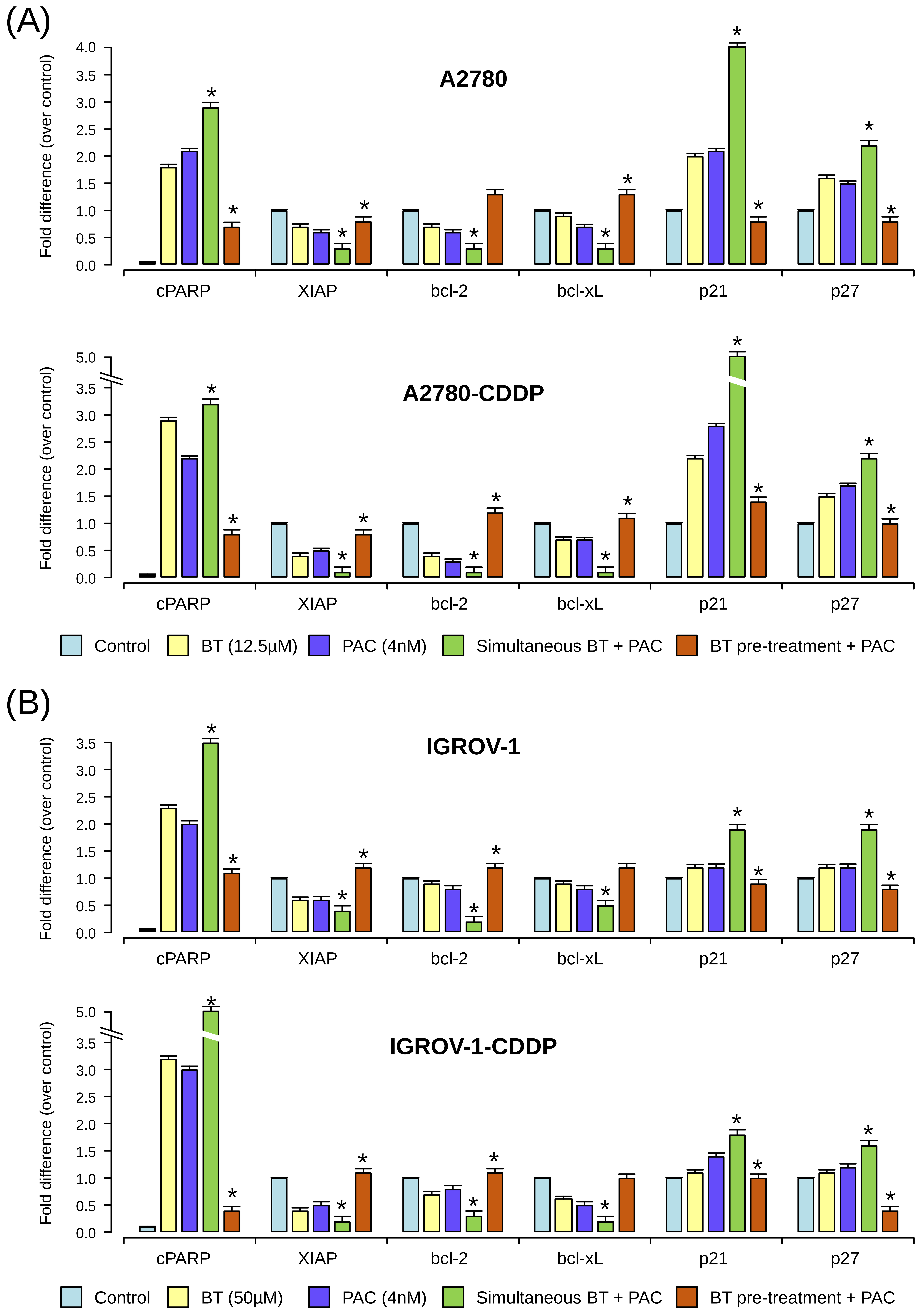

S2 Fig. Western blot quantification of various apoptotic and cell cycle regulatory molecules presented in Figs. 5C **(A)** and 5D **(B)**. Data represent fold difference over control cells (vehicle treated only). Values are means ± S.E.M. of three independent experiments. Asterisks (*) denote significant difference, at $P < 0.05$, as compared to cells treated with paclitaxel alone.
